# Gene dosage of independent dynein arm motor preassembly factors influences cilia assembly in *Chlamydomonas reinhardtii*

**DOI:** 10.1371/journal.pgen.1011038

**Published:** 2024-03-18

**Authors:** Gervette M. Penny, Susan K. Dutcher

**Affiliations:** Department of Genetics, Washington University in Saint Louis, Saint Louis,Missouri, United States of America; Rutgers University New Brunswick, UNITED STATES

## Abstract

Motile cilia assembly utilizes over 800 structural and cytoplasmic proteins. Variants in approximately 58 genes cause primary ciliary dyskinesia (PCD) in humans, including the dynein arm (pre)assembly factor (DNAAF) gene *DNAAF4*. In humans, outer dynein arms (ODAs) and inner dynein arms (IDAs) fail to assemble motile cilia when DNAAF4 function is disrupted. In *Chlamydomonas reinhardtii*, a ciliated unicellular alga, the *DNAAF4* ortholog is called *PF23*. The *pf23-1* mutant assembles short cilia and lacks IDAs, but partially retains ODAs. The cilia of a new null allele (*pf23-4*) completely lack ODAs and IDAs and are even shorter than cilia from *pf23-1*. In addition, PF23 plays a role in the cytoplasmic modification of IC138, a protein of the two-headed IDA (I1/f). As most PCD variants in humans are recessive, we sought to test if heterozygosity at two genes affects ciliary function using a second-site non-complementation (SSNC) screening approach. We asked if phenotypes were observed in diploids with pairwise heterozygous combinations of 21 well-characterized ciliary mutant *Chlamydomonas* strains. Vegetative cultures of single and double heterozygous diploid cells did not show SSNC for motility phenotypes. When protein synthesis is inhibited, wild-type *Chlamydomonas* cells utilize the pool of cytoplasmic proteins to assemble half-length cilia. In this sensitized assay, 8 double heterozygous diploids with *pf23* and other *DNAAF* mutations show SSNC; they assemble shorter cilia than wild-type. In contrast, double heterozygosity of the other 203 strains showed no effect on ciliary assembly. Immunoblots of diploids heterozygous for *pf23* and *wdr92* or *oda8* show that PF23 is reduced by half in these strains, and that PF23 dosage affects phenotype severity. Reductions in PF23 and another DNAAF in diploids affect the ability to assemble ODAs and IDAs and impedes ciliary assembly. Thus, dosage of multiple DNAAFs is an important factor in cilia assembly and regeneration.

## Author summary

Motile cilia are essential for the movement of cells and fluids. In humans, motile cilia defects cause primary ciliary dyskinesia (PCD), a rare, recessive disease characterized by recurrent respiratory infections, left-right asymmetry defects, ear infections, and infertility. A subset of PCD genes encodes proteins that function in the cytoplasm of the cell to assemble the dynein arms. *Chlamydomonas reinhardtii*, a single-celled alga, provides a model for understanding genetic causes of PCD. We asked if heterozygosity at two PCD genes causes cilia motility or assembly defects. In 211 double heterozygotes tested, we observed no motility defects. A unique feature of *Chlamydomonas* is that the cilia can be easily removed by pH shock; this is followed by rapid cilia assembly. When protein synthesis is inhibited after the pH shock, cells utilize the cytoplasmic pool of proteins and regrow shorter cilia. Eight of the double heterozygotes that have at least one mutation in a cytoplasmic dynein assembly gene assemble even shorter cilia than wild-type cells. These results suggest that a change in quantities of multiple cytoplasmic dynein assembly proteins is an important factor in cilia assembly.

## Introduction

Motile cilia are microtubule-based organelles that generate fluid flow or provide cellular motility [1]. In humans, pathogenic variants in 58 genes, to date, cause a rare disease called primary ciliary dyskinesia (PCD), characterized by recurrent lung infections, neonatal respiratory distress, bronchiectasis, otitis media, situs inversus/ambiguus, and male infertility [2–6]. *Chlamydomonas reinhardtii*, a haploid, single-celled, photosynthetic alga, is used extensively for the study of motile cilia due to its ease of genetic, biochemical, and microscopic analyses. *Chlamydomonas* has two cilia that are 10–12 μm long that it uses to swim and mate. The cytoskeleton of the cilia (or axoneme) is composed of 9 outer microtubule doublets (MTDs) and two single microtubules known as the central apparatus. A unique feature of *Chlamydomonas* is that cilia can be removed by pH shock; they then reassemble to full-length within 90 min. This requires both the pool of proteins that exists in the cytoplasm after deciliation, and *de novo* protein synthesis [7]. This *de novo* synthesis occurs following a transcriptional upregulation of genes that begins immediately after deciliation [8,9]. When new protein synthesis is blocked by the addition of cycloheximide, a protein synthesis inhibitor, wild-type haploid cells only assemble half-length cilia. In *pf16*, a central apparatus mutant, the regrown cilia were only about 2 μm long [7]. In addition to *pf16*, several other central apparatus proteins were missing from the *pf16* mutant cilia [10–12]. The absence of multiple proteins when one motile cilia gene is mutated is also observed for mutants that affect most other ciliary structures [13–15]. These studies highlight two important points. First, the total quantity of proteins available in the cytoplasm is a limiting factor in cilia assembly. Secondly, the loss of one protein necessary for assembly of a ciliary substructure can affect other proteins in the pool.

The dynein arms are essential for cilia motility. The outer dynein arms (ODAs) are attached to the ciliary axoneme every 24 nm. They generate the force that leads to ciliary bending through sliding and determine the beat frequency of the cilia [16–18]. In *Chlamydomonas*, ODAs are large megadalton complexes comprised of 3 heavy chains [19–22], 2 heterodimer-forming intermediate chains [23–26], and 11 light chains [27]. The inner dynein arm (IDA) motors create the asymmetric breaststroke waveform that results in forward swimming [16]. There are 7 IDAs that each repeat every 96 nm. IDA I1/f has 2 heavy chains, 3 intermediate chains, several light chains, and FAP120. The remaining 6 IDAs have one heavy chain and various combinations of other proteins including light chains [27]. ODAs and IDAs are present on all 9 doublets, except MTD1 that lacks ODAs [28,29]. Therefore, to build two full-length functional cilia, approximately 24,000 ODA and 16,000 IDA heavy chains (for a 12 μm cilium) must be translated, folded and assembled with the other dynein subunits [30]. Furthermore, the ODA heavy chains are over 4000 amino acids long and take approximately 15 min to translate. Since a full-length cilium can be assembled in approximately 90 min, this implies that there is a large biosynthetic demand placed on the cell that needs to be fulfilled in a short period of time [31]. Loss of ODAs, IDAs, and other axonemal proteins, and the factors needed to assemble or transport them results in the formation of dysfunctional cilia, or the absence of cilia. Haploid strains with mutations that result in the simultaneous loss of both ODAs and IDAs assemble short cilia in *Chlamydomonas*. In mutants that are missing only ODAs or IDAs, the cilia are full-length. Therefore, we reasoned that a change in dosage of multiple ODA and IDA proteins could hinder the cell’s ability to assemble functional dynein motor complexes, and therefore limit cilia function or assembly.

Genetic analyses identified proteins required for the proper assembly of ODAs and IDAs known as dynein arm preassembly factors, or DNAAFs [32–34]. DNAAFs localize primarily to the cytoplasm, although there are a few exceptions [35–38]. DNAAFs are a group of structurally diverse proteins that interact with chaperone complexes to guide the folding and stabilization of ODAs and IDAs. They have many different domains that include ones involved in protein-protein interactions and scaffolding functions. For example, PF23 contains several TPR repeats, structures that are well known for their scaffolding functions in other proteins [39,40]. Yeast two-hybrid assays and co-immunoprecipitation of recombinant proteins show that PF23 forms a large scaffolding hub with other preassembly factors that include MOT48, RPAP3, WDR92, FBB18, and ODA7 [41]. RUVBL1/2, a member of the R2TP co-chaperone complex also interacts with this hub of preassembly factors [42]. Co-immunoprecipitation with GST and His-tagged proteins in human cell lysates show that DNAAF2 (PF13), a PIH-domain-containing protein also interacts with DNAAF4 (PF23) [43]. In addition, PF13 forms part of a non-canonical R2TP complex that is involved in many cellular processes [44,45]. In addition to interacting with several DNAAFs, the WDR (tryptophan-aspartic-repeat) protein WDR92 (Monad) is a part of multiple co-chaperone complexes including R2TP and prefoldins [45,46]. A different group of proteins involved in dynein assembly are called ‘maturation’ factors. One of the maturation factors, ODA8, is important for efficient preassembly of ODA heavy chains and preparing outer dynein arms to be axoneme-binding competent [47,48]. Other groups of proteins important for ODA and IDA transport and attachment to the ciliary axoneme include the intraflagellar transport (IFT) trains, the ODA and IDA adapters, and the ODA docking complex [15,49–52].

The analysis of DNAAFs suggests a complex, non-linear role in dynein preassembly. The order of ODA and IDA preassembly and the role that each DNAAF plays in dynein preassembly remains largely unknown. Importantly, existing data shows the co-operativity of DNAAFs and chaperone protein complexes throughout the dynein preassembly process. As in humans, mutations in *DNAAF* genes in *Chlamydomonas* cause a loss of ODAs and IDAs in cilia. Unlike in humans, the cilia are short; this resembles the phenotype observed in double mutants that lack both ODAs and IDAs. In the cytoplasm of some *DNAAF* mutants, the stability and abundance of some dynein heavy chains are affected [34,46,53,54]. These observations suggest that proper folding and stabilization of ODAs and IDAs is necessary to supply the cytoplasmic pool with sufficient dynein complexes for cilia assembly.

One technique to study genetic interactions and identify protein products that interact or belong to the same pathway is second-site non-complementation (SSNC). The complementation test is based on the premise that two recessive mutant alleles belong to different genes if they produce a wild-type phenotype when each allele is in *trans* (on different parental chromosomes) [55]. If the two heterozygous mutant alleles produce a mutant phenotype when in *trans*, they exhibit a failure to complement and are presumed to belong to the same gene. SSNC occurs because there is failure to complement when the alleles are in different genes [56]. One mechanism of SSNC is through gene dosage, when two genes are heterozygous for null alleles. In this case, the cell will only produce one-half the amount of each gene product, which is insufficient for normal cellular function [56,57].

In this study, we utilized well-characterized haploid *Chlamydomonas* motile cilia mutant strains from the *Chlamydomonas* Resource Center to conduct a SSNC screen for motility and cilia phenotypes [58,59]. These strains originated from multiple large screens for mutants that are slow-swimming, paralyzed, or have short cilia [17,29]. These mutants were used to generate diploid strains. The motility of these diploid strains showed no obvious swimming phenotype. Therefore, a sensitized screen was employed to determine whether cilia length phenotypes would be observed. The strains were deciliated and allowed to regenerate in cycloheximide. Because *de novo* protein synthesis is inhibited, the cell is forced to utilize the existing protein pool to assemble cilia. This sensitizes the cells to changes in the protein levels in the cytoplasmic protein pool. We found that structural proteins like ODA and IDA heavy chains do not show length phenotypes in double heterozygotes that have lost one allele of each gene. These genotypes do not affect cilia regeneration. In contrast, diploids heterozygous for DNAAFs show sensitivity to reduced quantities of these proteins and regenerate cilia that are shorter than wild-type. In addition, multiple SSNC combinations involve the preassembly factor PF23. Our methodology supports the idea that PF23 is a hub protein and acts at multiple steps in the dynein preassembly pathway. The assembly of dynein arms is impacted by the concentration of preassembly factors.

## Results

### Motile cilia mutants in *Chlamydomonas* have diverse phenotypes

**Fig 1:**
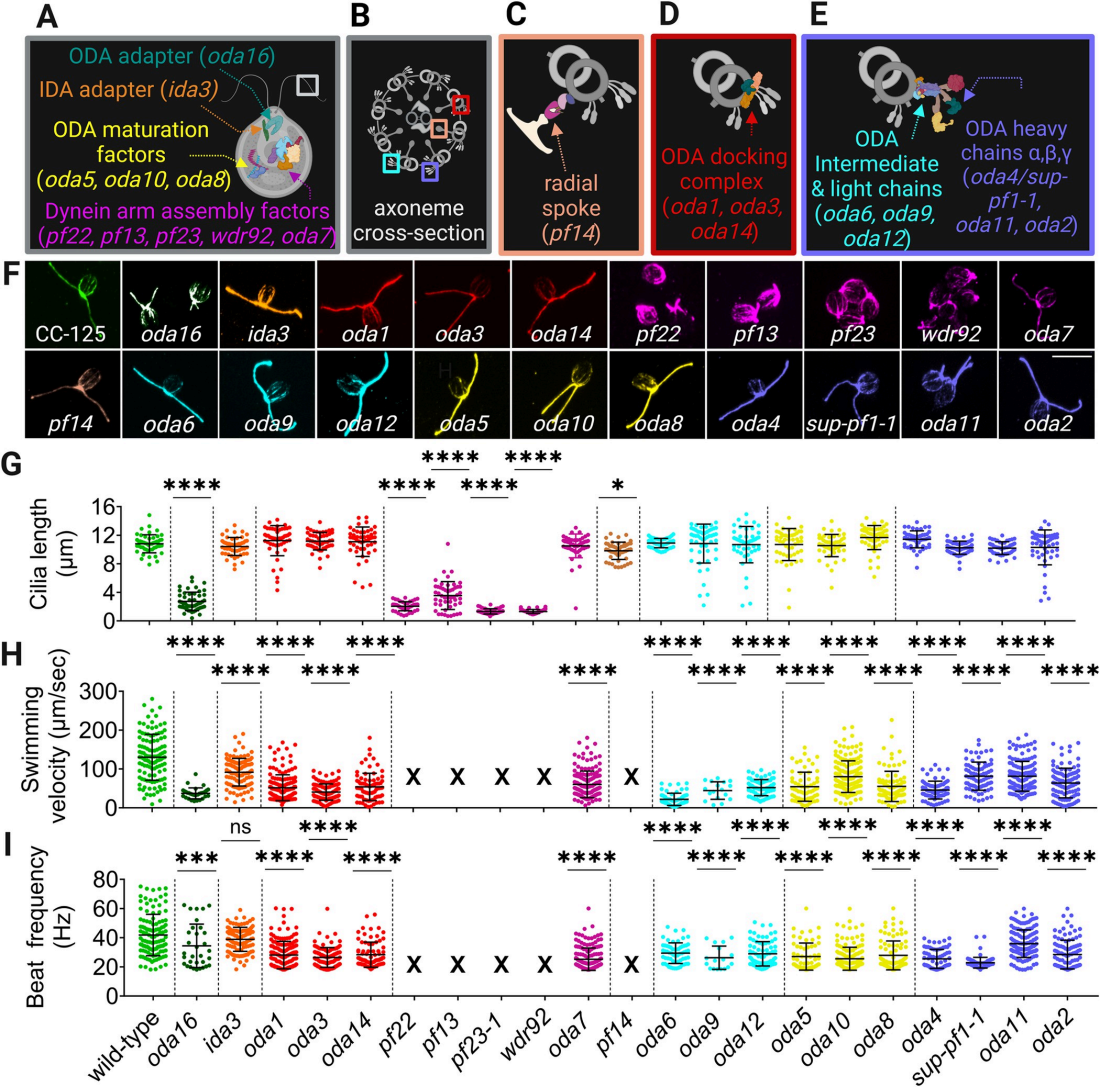
Different classes of motile cilia genes and dynein preassembly factor mutants have diverse phenotypes. (A-E) Motile cilia genes in this study are grouped according to their function or the structure affected. The mutant strains in each group are listed in parentheses. (A) Dynein arm preassembly factors (pink), ODA maturation factors (yellow), ODA adapter (dark green) and IDA adapter (orange) proteins. (B) A cross-section of the *Chlamydomonas* axoneme outlined by the white box in (A). The boxes highlight the locations of four structural groups; they are the radial spoke (tan, C) the ODA docking complex (red, D), and the ODA intermediate and light chains (cyan, E) and ODA heavy chains (purple, E). (F) Wild-type *Chlamydomonas*, and mutants from each functional group in A-E, are stained with an antibody against acetylated α-tubulin to show the cilia, basal body, and rootlet microtubules. (G) Quantification of cilia lengths. One cilium from 50 cells were measured for each strain. Statistical analysis was performed using a one-way ANOVA with adjustments for multiple comparisons. Each strain is compared to wild-type strain CC-125. (H) Swimming velocity measurements for each strain in F. (I) Beat frequency measurements for each strain in F. Strains *pf22*, *pf13*, *pf23-1*, *wdr92* and *pf14* are non-motile, indicated by an ‘X’. ‘*’ p < 0.05; ‘**’ p < 0.001; ‘***’ p < 0.0001; ‘****’ p < 0.00001; ‘ns’ not significant. Created with BioRender.

To study how double heterozygous mutations affect cilia assembly and function in *Chlamydomonas* by using a SSNC screening method, we selected 21 strains with mutations that alter the assembly of the outer and/or inner dynein arms or their transport into cilia (Fig 1A–1F). To verify the phenotypes of the strains, we examined cilia length, swimming velocity, and beat frequency of each mutant (Fig 1G–1I, S4 Table). Immunofluorescence staining with an antibody to acetylated α-tubulin that stains cilia, basal bodies, and rootlet microtubules confirms that strains with preassembly factor mutations (*pf22-1*, *pf13-1*, *pf23-1*, and *wdr92-2*) have short, immotile cilia (Fig 1F–1I). One exception is the preassembly factor mutant *oda7* that assembles full-length cilia and is motile (Fig 1F–1I). Like the other *oda* strains, the *oda7* cells assemble full-length cilia and generally swim at about one-half the wild-type speed, with a reduced beat frequency [18,60]. Most of the *oda16-1* cells were immotile, but approximately 10% of cells swam slowly. Non-motility in *oda16* mutants was not reported in earlier studies [50,61]. However, many cells showed a slow motility phenotype as gametes. The *ida3* strain swims more slowly than wild-type but has a wild-type beat frequency and cilia length (Fig 1F–1I) [51]. These strains show distinctive and easily observable phenotypes. If SSNC occurs, we hypothesized that it should be easy to identify double heterozygous strains with mutant phenotypes that resemble the parental phenotypes.

### Five unknown causative mutations were identified by whole genome re-sequencing

The type of allele or mutation present in a strain can influence how SSNC occurs [57]. The known causative genes and mutations in most strains were identified by rescue with a transgene or by sequencing (Table 1). The causative mutations in five strains (*pf13-1*, *oda3-1*, *oda4-1*, *oda9*, and *oda11-1*) were unknown. Therefore, we used whole genome short-read re-sequencing [62]. The mutations in strains *oda3-1*, *oda9*, and *oda11-1* were identified (Tables 1, S1 and S5). The SnpEff analysis pipeline [63] did not reveal any candidate mutations in *pf13-1* and *oda4-1*. After manual curation of sequence from the *pf13-1* strain, we identified a 44.7 kb deletion that removes the *PF13* gene and 6 other genes (Tables 1 and S1, S1A–S1B Fig). PCR shows that the centromere-adjacent breakpoint removes all the exons of *PF13* (exons 3, 7 and 10 were tested by PCR, S1 Table), but the 3’UTR is retained. The telomere-adjacent breakpoint involves Cre09.g411700, which contains 3 exons and is the last gene disrupted by the deletion. Only a small part of the 3’ end of exon 3 is deleted; exons 1 and 2 are retained (S1B Fig). The *pf13-1* mutation was rescued by a transgene containing the wild-type *PF13* gene that was generated by recombineering (S2 Fig) [64,65]. Thus, the deletion of *PF13*, but not the other 5 genes, causes the phenotype observed in *pf13-1*. The reads at the *ODA4* (*DHC14*) locus were manually examined. We found a 5 base-pair deletion in the *oda4-1* mutant that changes the reading frame in one of the AAA ATPase domains (S1 Table, S1C Fig). PCR analysis confirmed that the sequence changes identified by resequencing of *oda3-1*, *oda4-1*, *oda9*, and *oda11-1* are present in the mutant strains (S1 Table). Most of the mutant strains used are null or predicted null except for *pf23-1* and *sup-pf1-1*. We hypothesized that possible phenotypes observed due to SSNC in double heterozygotes would be due to dosage effects from the loss of one allele at each locus in strains with null alleles.

**Table 1:** *Chlamydomonas* mutants used to generate diploids for SSNC screening.

Category	Mutant	Human Ortholog	PCD Gene	DNA or animo acid change	Type of allele	Ref.
**Dynein arm assembly factor**	*pf22-1*	*DNAAF3*	Yes	W79X	Null	[66]
	*oda7-1*	*DNAAF1*	Yes	Exon 1 insertion	Null	[67]
	***pf13-1***	*DNAAF2*	Yes	49 kb deletion	Null	[68]
	*pf23-1*	*DNAAF4*	Yes	In-frame exon 5 deletion	Altered**	[43, 69]
	*pf23-4*	*DNAAF4*	Yes	Insertion exon 1	Null	This work
	*wdr92-2* ^*X*^	*DNAAF10*	No	Exon 5 deletion, insertion	Null	[46]
**Accessory complex**	*oda8*	*LRRC56*	Yes	E36fs	PN	[47]
	*oda5*	*CCDC63*	No	V108X	Null	[70, 71]
	*oda10*	*ODAD3*	Yes	Unknown	Unknown	[71, 72]
**ODA docking complex**	*oda1*	*ODAD1*	Yes	E46X	Null	[52]
	***oda3-1***	*ODAD3*	Yes	M279fs; 1bp deletion	Null	[15]
	*oda14*-*F28*	None	No	Exon 1 insertion	Null	[73]
**ODA intermediate & light chain**	*oda6-95*	*DNAI2*	Yes	V54Gfs	Null	[23, 74]
	***oda9***	*DNAI1*	Yes	K225fs	Null	[75, 76]
	*oda12-2*	*DYNLT2*	No	3’UTR insertion	Null	[77]
**ODA heavy chain**	***oda4-1***	*DNAH9/11/17*	Yes	R2872fs; 5 bp deletion in exon 22	Null	[76]
	*sup-pf1-1* ^*+*^	*DNAH9/11/17*	Yes	21bp in-frame deletion in exon 25 of *ODA4*	Altered**	[78]
	***oda11-1***	None	Yes	W778X	Null	[19]
	*oda2-921* ^*X*^	*DNAH5/8*	Yes	Exon 9 insertion	Unknown	[59]
**ODA adapter** **IDA adapter**	*oda16-1*	*DAW1*	No	Insertion unknown	Null	[61]
	*ida3*	None	No	W22X	Null	[51]
**Radial spoke**	*pf14*	*RSPH3*	Yes	Q21X	Null	[14]

Mutations are organized by the known structural or functional group. Strains re-sequenced in this work are in bold.

**These strains produce an altered, shortened protein with partial function.

^X^CLiP strain [60].

^+^ The *sup-pf-1-1* mutation is an allele of *ODA4* that alters the microtubule-binding stalk region of the ODA β-HC.

### A null *Chlamydomonas pf23-4* mutant lacks ODAs and IDAs in the cilia

**Fig 2:**
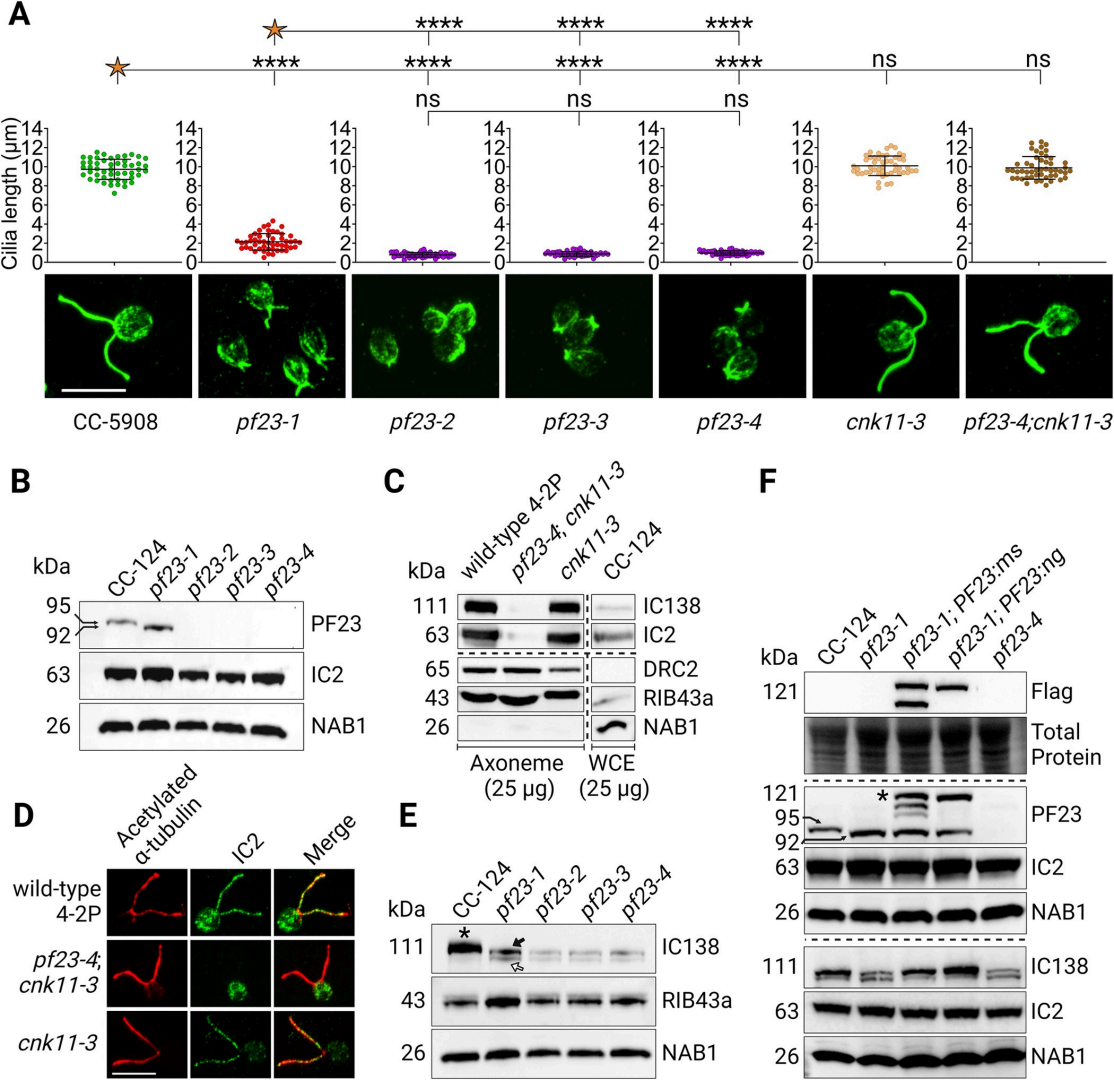
Characterization of null *pf23* alleles generated by CRISPR/Cas9 site-directed insertional mutagenesis. (A) Immunofluorescence staining of gametic cells using an antibody to acetylated α-tubulin to visualize cilia length of wild-type, *pf23-1*, and three CRISPR-generated *pf23* strains. The quantification of length for each strain is positioned directly above its corresponding image. (B) Immunoblot probing gametic whole cell extracts for PF23 and IC2 intermediate chain proteins that shows that these CRISPR-generated alleles do not produce PF23 protein and are null. The cytoplasmic protein NAB1 is used as a loading control. (C) Immunoblot of isolated cilia of wild-type, *pf23-4; cnk11-3*, *cnk11-3*, and wild-type CC-124 for various axonemal components. IC138 and IC2 are missing in *pf23-4; cnk11-3* axonemes. NAB1 is used to assess cytoplasmic contamination. The NAB1 image shown is overexposed to show the relative abundance in CC-124 whole cell extracts compared to its absence in axonemes. (D) Immunofluorescence of strains in (C) using an antibody against IC2 shows that IC2 is absent in *pf23-4; cnk11-3* cilia. (E) Immunoblot of *pf23* mutant strains for IC138 I1/f intermediate chain and MIP RIB43a. The wild-type IC138 band in CC-124 (indicated by an asterisk) is slightly larger than the two bands observed in the *pf23-1* (upper band: black arrow, lower band: white arrow) and *pf23* null mutant strains. NAB1 is used as cytoplasmic loading control. (F) Immunoblot of wild-type CC-124, *pf23-1*, *pf23-4*, and two independent rescue strains *pf23-1; PF23*:*ms-TG* and *pf23-1; PF23*:*ng-TG* (ms: m-scarlet-3x FLAG, ng: neon green-3x FLAG, TG: transgene). Top: anti-FLAG antibody shows the expected size for PF23 protein with m-scarlet or neon-green plus 3x FLAG. Middle: probing with anti-PF23 antibody shows rescue of PF23 wild-type protein expression in both transgenic strains in addition to the expression of the shortened version of PF23. The asterisk indicates the correct band that corresponds to tagged PF23 protein at the expected size. Bottom: The IC138 altered mobility observed in *pf23-1* is restored to wild-type in both rescue strains. (C, F) The horizontal dashed lines indicates individual gels with the same samples. The vertical line in (C) indicates that the CC-124 sample was on the same gel, but the order of the lanes were rearranged. Statistical analysis conducted using a one-way ANOVA with corrections for multiple comparisons. ‘*’ p < 0.05; ‘**’ p < 0.001; ‘***’ p < 0.0001; ‘****’ p < 0.00001; ‘ns’ not significant. Created with BioRender.

The *Chlamydomonas pf23-1* allele contains an in-frame deletion of exon 5 and surrounding introns that produces a smaller PF23 protein with reduced function. Spectral counting of heavy chain bands from *pf23-1* showed almost complete loss of the IDA proteins, while ODA species were reduced by approximately 50–60% [69]. We generated a null *pf23* allele to determine whether complete loss of PF23 would have greater effects on dynein preassembly. CRISPR/Cas9 site-directed insertional mutagenesis was used to insert the *aphvii* cassette that confers hygromycin resistance into exon 1 of the *PF23* gene [79] in the wild-type strain CC-5908. Three independent strains from two separate transformations were obtained and named *pf23-2*, *pf23-3*, and *pf23-4* (S3 Fig) [79]. Immunofluorescence analysis with an antibody to acetylated α-tubulin shows that gametic wild-type cells grow cilia 9.72 +/-1.06 μm long. The *pf23-1* strain assembles 2.14 +/- 0.86 μm long cilia, while the *pf23-4* null strain has cilia that are 0.87 +/- 0.23 μm (Fig 2A, S6 Table). Immunoblot analysis using an anti-PF23 antibody [46] shows that the CRISPR insertional mutants completely lack the PF23 protein, unlike the *pf23-1* strain that produces a smaller PF23 protein (Fig 2B). The complete loss of PF23 protein results in a more severe phenotype than in the pf23-*1* strain.

Previous meiotic analysis of all three strains showed that *pf23-2* and *pf23-3* produced meiotic nonviability, while crosses with *pf23-4* produced primarily tetrads with four viable progenies. Long-range sequencing of *pf23-2* and *pf23-3* shows that these strains contain translocations between the *PF23* gene and chromosomes 5 and 3, respectively [79]. Because *pf23-4* did not show any meiotic abnormalities, it is unlikely that it possesses a translocation. Therefore, we used this strain for further analyses.

Mutations in *CNK11*, a NIMA-related kinase, partially suppresses the cilia length defects of other mutant strains without rescue of the motility defects [80,81]. The *pf23-4* short cilia phenotype is rescued by *cnk11-3* (Fig 2A). Immunoblot analysis of cilia from *pf23-4; cnk11-3* confirms that IC138 and IC2 are missing from *pf23-4*, and the N-DRC component DRC2 and microtubule inner protein RIB43a are unchanged (Fig 2C). Immunofluorescence analysis also shows that IC2 fails to localize to the cilia in the *pf23-4* null strain (Fig 2D). These data confirm that the *pf23-4* allele shows a more severe assembly defect than *pf23-1*. ODAs and IDAS are affected but other structural proteins are not.

To further characterize the effects of the *pf23-4* null allele on assembly of dynein arms, isolated axonemes were collected. Mass spectrometry analysis of isolated cilia from the *pf23-4; cnk11-3* strain reveals that the ODA and IDA heavy, intermediate chains, and shared light chains are severely reduced or completely absent (Table 2, red), except LC4 and LC8 that is only mildly reduced (light green). LC8 is also found in the radial spokes. The light chain components of the monomeric IDAs responded differently to loss of PF23. For example, p28, which is in IDA a, c, and d, is completely missing. Actin is present in all the monomeric IDAs and is severely reduced. Centrin is present in IDA b, e, and g, but is unaffected. The p44 and p38 light chains are only found in IDA d. However, p44 is unaffected, while p38 is drastically reduced. DHC12, a minor IDA heavy chain, is missing from our wild-type proteomic data and could not be analyzed. The ODA docking complex proteins are unaffected. Components of other axonemal proteins including Modifier of Inner Arms (MIA), radial spokes, N-DRC, and the central apparatus remain largely unaffected, or only mildly reduced (green). The I1/f tether component FAP44 was unchanged. Interestingly, the tether-head protein FAP43 is retained, while its paralog FAP244 is lost. The *cnk11-3* mutation had little effect on the protein composition of the cilia (S2 Table).

**Table 2:** Mass spectrometry of axonemes from *pf23-4; cnk11-3*.

Protein	Ratio of *pf23-4; cnk11-3* to wild-type	Protein	Ratio of *pf23-4; cnk11-3* to wild-type
**Tubulin**		**Monomeric IDAs**	
Tubulin α	2.2	DHC9	0.0008
Tubulin β	1.1	DHC11	-
**ODA**		p28	-
α-HC	-	actin	0.009
β-HC	-	centrin	1.0
γ-HC	-	p44	1.0
IC1	-	p38	0.30
IC2	-	DHC12	NP
LC1	-	**IDA-associated**	
LC2	0.20	FAP44	3.1
LC3	0.002	FAP244	0.002
LC4	0.76	FAP43	3.3
LC5	-	CCDC146	4.1
LC6	0.01	FAP57	3.6
LC9	0.002	FAP263	0.42
LC10	-	**ODA Docking Complex**	
**ODA and IDA light chains**		DC1	1.7
LC7a	0.033	DC2	2.1
LC7b	-	DC3	1.8
LC8	0.8	**Modifier of Inner Arms**	
**IDA I1/f**		FAP73	1.8
I1/f α	-	FAP100	1.3
I1/f β	-	**Radial Spoke**	
IC138	-	FAP61	10.1
IC140	-	FAP91	4.1
IC97	-	FAP184	1.6
FAP120	-	**Nexin-Dynein Regulatory Complex**	
TCTEX1	0.07	DRC1	3.1
TCTEX2b	0.23	DRC2	2.0
**Monomeric IDAs**		**Central Apparatus**	
DHC2	0.002	HYDIN	3.6
DHC3	-	CPC1	0.40
DHC4	0.006	**Microtubule Inner Protein**	
DHC5	0.0004	FAP166	2.3
DHC6	0.001	RIB72	4.0
DHC7	-	RIB43a	4.2
DHC8	0.002	PACRG	2.6

The relative abundance of peptides from axonemes of *pf23-4*; *cnk11-3* strains normalized to wild-type axonemes is shown. Ratios are obtained from the normalized intensities (S2 Table). A row with a hyphen (-) or red shading indicates that the peptide was undetectable or extremely low compared to wild-type preparations. Rows with yellow shading are moderately decreased, and rows with green shading are close to, or at wild-type levels. Blue shading indicates that this protein was not identified in the wild-type samples and could not be analyzed. NP: not present. Corresponding gene and mutant names for each protein is indicated in S2 Table.

### Preassembly factor PF23 is involved in cytoplasmic IC138 modification

Immunoblots of whole cell extracts of *pf23-1*, *pf23-2*, *pf23-3*, and *pf23-4* haploid strains with an antibody against the IDA I1/f intermediate chain, IC138, show two bands in whole cell extracts instead of the single band found in wild-type strains. Both bands migrate faster than the band in wild-type (Fig 2E). We transformed *pf23-1* with a wild-type copy of *PF23* with a fluorescent tag and 3x FLAG tag (S4 Fig). In two independent rescued strains, the IC138 protein is restored to the wild-type pattern (Fig 2F). Since there is no IC138 present in the proteomic analysis of *pf23-4; cnk11-3* cilia (Fig 2C), the IC138 protein with altered mobility occurs exclusively in the cytoplasm of *pf23* mutants.

### Cilia length is independent of cell size in vegetative *Chlamydomonas* cells

**Fig 3:**
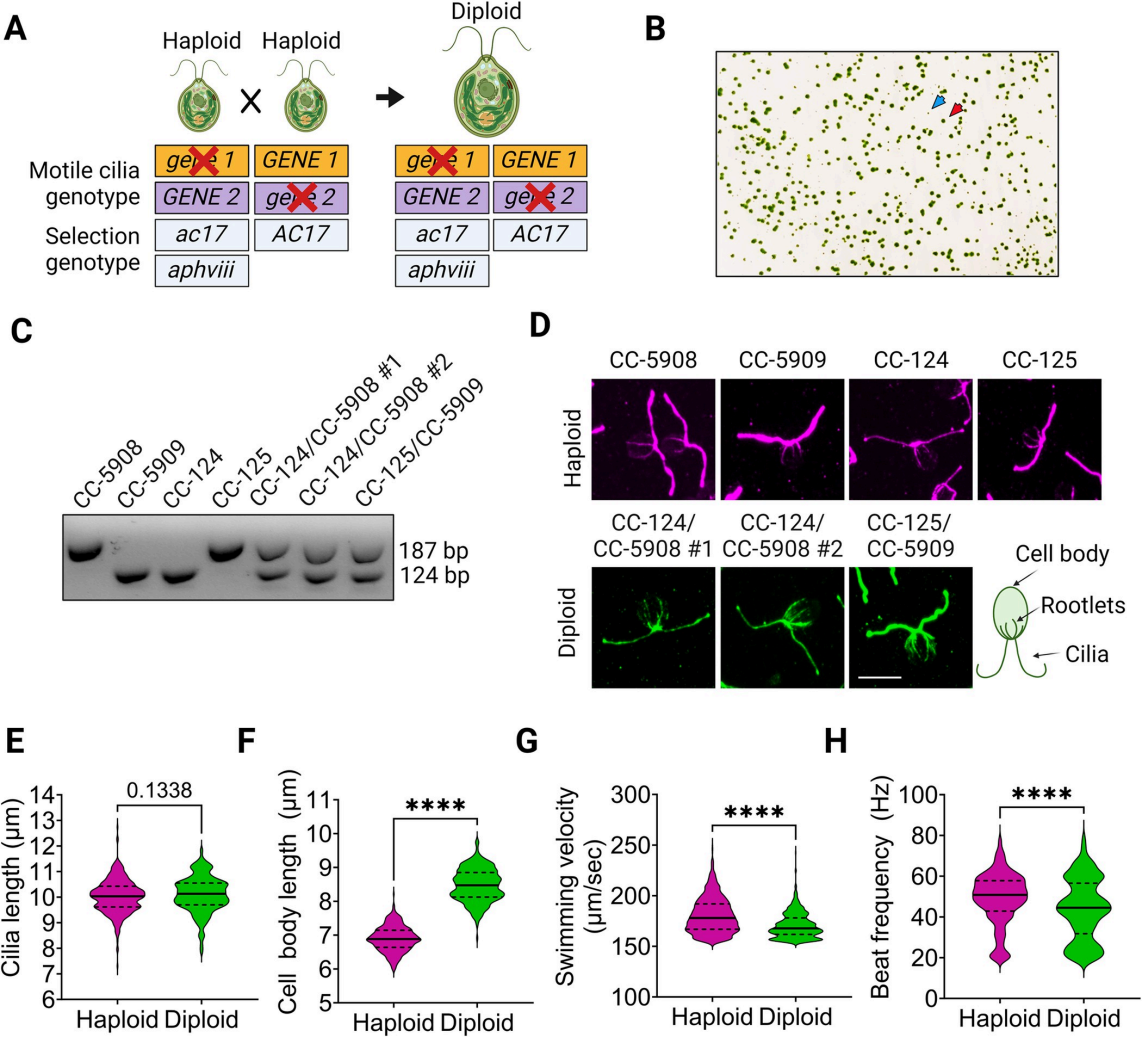
Generation and comparison of *Chlamydomonas* haploid and diploid strains. (A) Diploids were selected after 3–5 days on medium lacking acetate and containing paromomycin. Haploid parental strains with the genotype *ac17; aphviii* (CC-5908 and CC-5909) die on plates that lack acetate although they are resistant to paromomycin. Haploid parental strains with the genotype *AC17* grow in the absence of acetate and die on medium with paromomycin. Only diploid cells survive on plates lacking acetate and containing paromomycin. The two sets of haploid cells each carry mutations in two different cilia genes (*gene 1* or *gene 2*). Mating these strains produces diploid cells that are heterozygous for both mutations. (B) Colonies from diploid cells are bright green (red arrow) on a pale green background of haploid cells (blue arrow). (C) PCR amplicons for the *MTD* gene (mating-type minus with 124 bp product) and *MTA* gene (mating-type plus with 187 bp product) distinguish haploid and diploid strains. Diploid strains have both mating type loci. (D) Immunofluorescence of vegetative haploid strains with the genotype *ac17; aphviii (*CC-5908, CC-5909), CC-124 and CC-125 wild-type strains, and diploid strains generated by crosses between CC-5908 and CC-124 or CC-5909 and CC-125. Cells stained with an antibody against acetylated α-tubulin. An illustration of the cilia, rootlets and cell body is provided for reference. (E) cilia length, (F) cell body length measured from the apex of the cell to the base of the cilia of strains shown in D, (G) swimming velocity, and (H) beat frequency. The center line in each graph represents the median, while the top and bottom lines represent the upper and lower quartiles respectively. Statistical analysis for each graph was performed using a two-tailed t-test. ‘*’ p < 0.05; ‘**’ p < 0.001; ‘***’ p < 0.0001; ‘****’ p < 0.00001; ‘ns’ not significant. Created with BioRender.

To identify gene pairs that show SSNC between motile cilia mutants, the strains in Table 1 were used to generate 211 double heterozygous strains. To create a diploid that is heterozygous for two ciliary gene mutations, one haploid strain with a mutation in *gene 1* and wild-type for *GENE 2* was mated to a second haploid strain with a mutation in *gene 2* and wild-type for *GENE 1* (Fig 3A). Each strain contains a selectable genotype for either acetate auxotrophy (*ac17*) or paromomycin resistance (*aphviii*). The *ac17* mutation and *aphviii* transgene was obtained by crosses to strains CC-5908 and CC-5909 (see Materials and Methods). The selection for the *aphviii* gene was more stringent than the previously used *nit2* mutation [82]. The selectable genotypes are reciprocal in each haploid strain (Fig 3A). Diploids appear as bright green colonies on medium lacking acetate and containing paromomycin (Fig 3B). In addition, to verify the recessive nature of each allele, each strain was used to create 21 different single heterozygous diploids with one wild-type and one mutant copy of each allele. Wild-type diploids were also generated as controls.

We compared haploid and diploid wild-type *Chlamydomonas* vegetative cells. Previous analyses of diploid cells reported differences in cell size, mating-type, and DNA content compared to haploid cells [83]. Mating-type PCR analysis confirms that diploid cells contain both mating-type plus and mating-type minus loci (Fig 3C) [84,85]. Diploids generated in this work were also genotyped using PCR primers for the heterozygous selectable marker genes *ac17/AC17* and *aphviii* (S5 Fig). Diploid cells appear larger and swim more slowly than haploid cells by phase microscopy. To quantitate these characteristics, we performed immunofluorescence assays (Fig 3D) to compare cilia lengths; the cilia of diploid cells are similar in length to haploid cells (Fig 3D–3E). Diploid cell bodies are longer (8.47 +/- 0.48 μm) than haploids (6.89 +/- 0.37 μm) (Fig 3D, 3F, S7 Table); these data agree with previous results [83]. These data suggest that cilia length is regulated independently of cell size in vegetative cells. Diploid strains also exhibit slower swimming velocities and beat frequencies than haploid strains (Fig 3G–3H).

**Fig 4:**
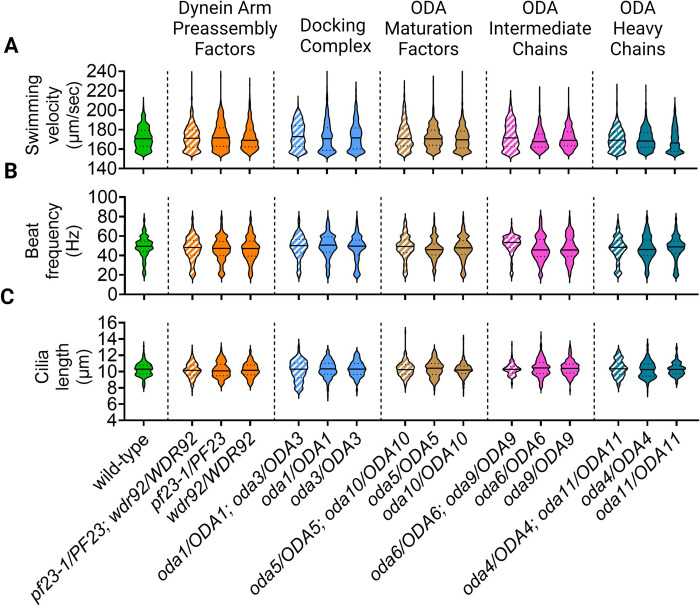
Motile cilia structural and dynein preassembly factor genes are recessive in vegetative diploids. A subset of diploids was examined for the occurrence of SNNC in 5 double heterozygous strains compared to single heterozygous controls. (A) swimming velocity, (B) beat frequency, and (C) cilia length for wild-type diploids and heterozygous diploids with mutations in different ODA preassembly or structural genes. Pooled data for 3 biological replicates are shown for each strain. N = 50 cilia from 50 cells for each replicate. Solid green fill indicates wild-type, and single heterozygotes are represented by various other solid fill colors. Striped fill indicates double heterozygotes. The solid center line in each plot represents the median. Dashed lines indicate the upper and lower quartiles. There was no statistical significance between wild-type and any of the single or double heterozygous diploids when compared using a one-way ANOVA with corrections for multiple comparisons. Created with BioRender.

Initial analysis showed that none of the heterozygous diploid strains display parental mutant phenotypes. We quantified cilia length, swimming velocity, and cilia beat frequency of a subset of vegetative single and double heterozygous diploids. There were no differences in these phenotypes among the wild-type and heterozygous diploids (Fig 4A–4C, S8 Table). Additionally, no obvious patterns of motility defects were detected in any double heterozygotes. This suggests that under vegetative steady-state conditions, all the genotypes in our heterozygous diploid strains are recessive and do not show SSNC.

### Cilia length and growth rate during assembly is dependent on cell size and the available cytoplasmic protein pool

Next, we asked whether the length and assembly of cilia in diploid cells would be different compared to haploids during regeneration when the demand for ciliary proteins from the cytoplasm is high and the cytoplasmic pool is limiting [8]. To accomplish this, we developed a sensitized assay based on earlier work that assessed the effect of cycloheximide on cilia regeneration in haploid cells. In the absence of cycloheximide, wild-type haploid cells assemble ~ 9 to 10 μm cilia. However, when 10 μg/ml cycloheximide is added following deciliation, the cilia regenerate to about 5 to 6 μm (half-length) [7]. We hypothesized that wild-type diploid cells would also regenerate approximately half-length cilia when subjected to the same conditions.

**Fig 5:**
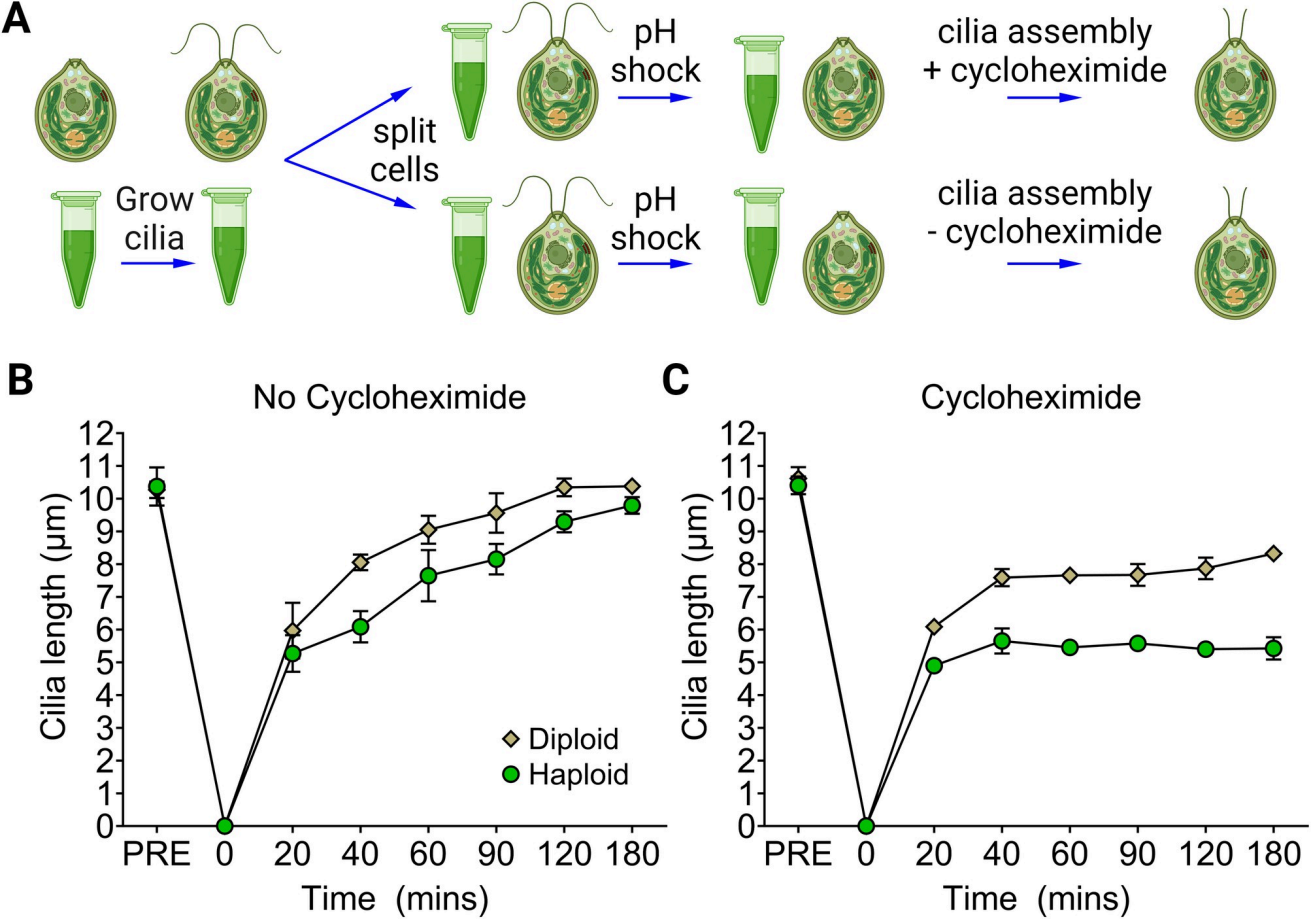
A larger cytoplasmic protein pool in diploids allows cilia to assemble longer than haploid cells. (A) Illustration of the protocol used for *Chlamydomonas* deciliation and cilia assembly. Vegetative haploid and cells were deciliated by pH shock and allowed to assemble for 180 min without (B) or with (C) 10 μg/ml cycloheximide added post-deciliation. Samples were collected for cilia length measurements at the timepoints shown. 100 individual cilia from 100 cells were measured for 4 wild-type haploid strains and 3 wild-type diploid strains. Error bars show the standard deviation of the means across the replicates for haploid or diploid cells. Created with BioRender.

Haploid and diploid wild-type strains were deciliated and allowed to assemble cilia in the absence or presence of cycloheximide (Fig 5A). In the absence of cycloheximide, diploid cells regrow cilia faster than haploid cells between 20 and 90 mins of regeneration. After 180 minutes, the haploid cell cilia, although slower to assemble, reached approximately the same terminal length as the diploid cells (Fig 5B, S9 Table). The final lengths of haploid and diploid cell cilia under regenerating conditions without cycloheximide are equivalent to the length before deciliation (Fig 3E).

The difference in cilia length between haploid and diploid cells in cycloheximide was measured. At 40 mins post-deciliation, diploid cilia are 7.58 +/- 0.26 μm long, while haploid cilia are 5.6 +/- 0.38 μm (Fig 5C, S9 Table). Furthermore, this length difference between the haploid and diploid cilia is not resolved after 180 mins. The cilia regeneration data in haploid cells recapitulate the observations of Rosenbaum *et al*. [7]. Surprisingly, our findings in diploid cells suggest that the cytoplasmic pool of proteins for building cilia is larger than in haploid cells since they assemble longer cilia and regenerate more quickly.

### Diploids heterozygous for mutations in *DNAAF*s show SSNC in a sensitized assay

In *Chlamydomonas*, SSNC phenotypes were observed in double heterozygous diploids of temperature-sensitive mutants of the intraflagellar transport (IFT) genes that encode the components of trains that move cargo into and out of the cilia (S3 Table, S6 Fig, [82]). Although the IFT proteins are not structural or dynein preassembly factors, their loss affects cilia length. The SSNC phenotypes are observed under restrictive temperature conditions. These data led us to hypothesize whether exposing other double heterozygous strains with mutations in different motile cilia genes to sensitizing conditions would yield SSNC phenotypes. The cytoplasmic pool is a limiting factor in cilia assembly, and diploid cells show reduced cilia assembly in the presence of cycloheximide. Previous experiments in haploid strains show that cilia are short in single mutants that affect preassembly or in double mutants of structural ODA and IDA genes [18,80,86,87]. Therefore, we hypothesized that heterozygous combinations of genes that affect both the ODAs and IDAs would show a phenotype under conditions that inhibit protein synthesis. It is this limiting pool of proteins that provides the sensitized background for our screen. It allows us to assay the effects of heterozygosity in one or two genes.

**Fig 6:**
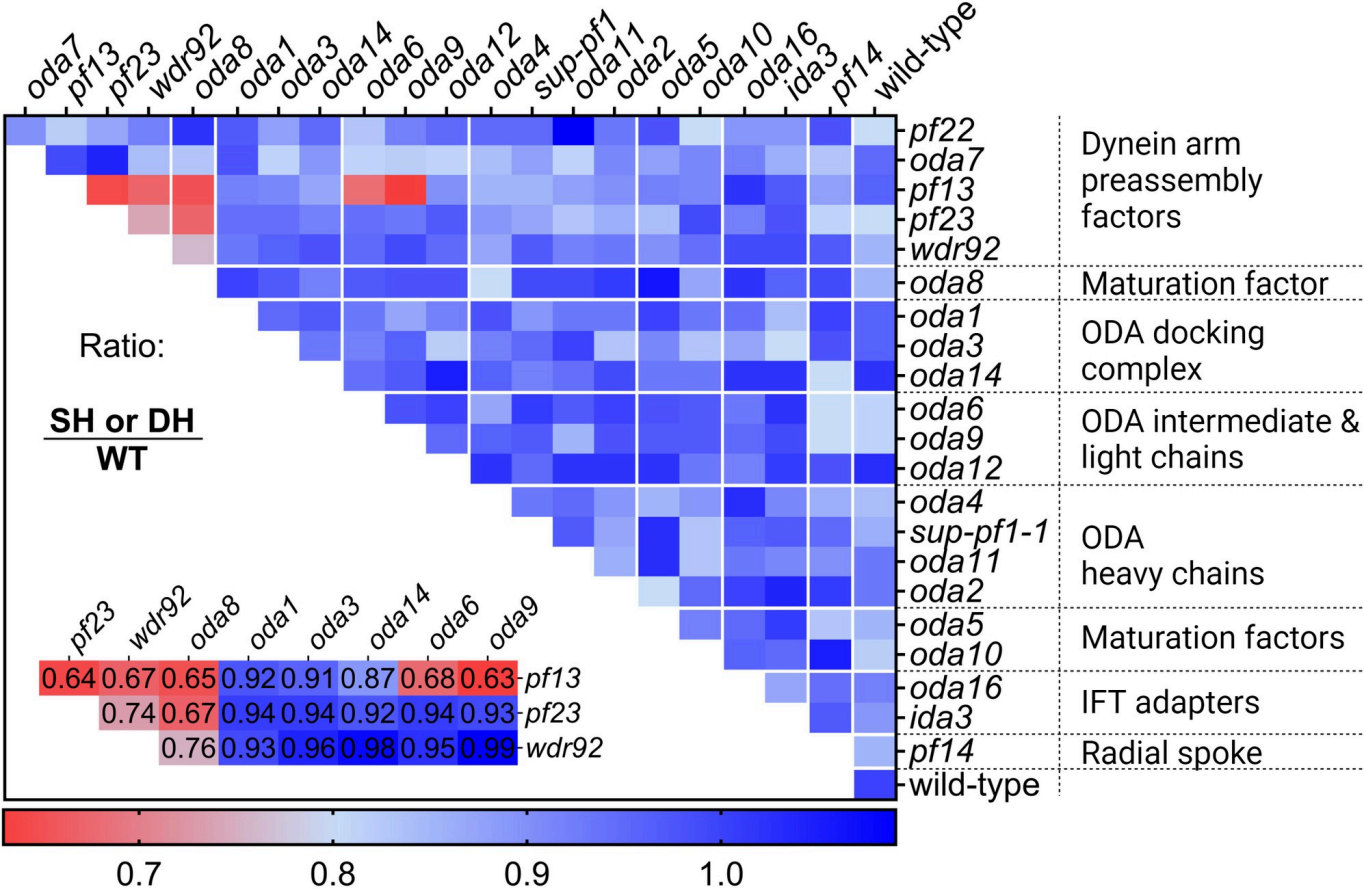
Cilia lengths following regeneration in cycloheximide for heterozygous diploids. Each strain and a wild-type control was deciliated and allowed to assemble cilia for 40 min in 10 μg/ml cycloheximide. Three biological replicates were measured for each heterozygous diploid. One biological replicate was measured for the wild-type diploid in each set of experiments. For each replicate, 100 individual cilia from 100 cells were measured and the average length was calculated. Each box shown in the heat map represents the product of the average length of the pooled biological replicates for each single (SH) (rightmost column) or double heterozygote (DH) divided by the average length of the wild-type diploid (WT). Cooler colors (blue) indicate that the cilia length of the tested heterozygotes is similar to wild-type cilia length. Warmer colors (red) indicate that cilia length is shorter than wild-type cilia length. An inset of the candidate hits in red is shown in the bottom left corner with the cilia length ratio value. Created with BioRender.

Using the protocol outlined in Fig 5A, the single and double heterozygous strains, and wild-type diploids were deciliated. Cycloheximide was added to limit the cytoplasmic pool during cilia regeneration. Cilia length was measured after 40 minutes. As shown in Fig 6 (right-most column) and S10 Table, all the mutations in the single heterozygous strains are recessive to the wild-type allele in this assay and support previous data from both human and model organism studies (Table 1). Double heterozygous strains with mutations in structural proteins (the radial spoke, the ODA structural proteins, and the ODA docking complex) all complement. The IFT adapters, as well as the maturation factors, also complement. These combinations do not show SSNC. In our screen, SSNC was observed only when at least one of the mutations was in a DNAAF. SSNC was observed between *pf13* and either *oda6* or *oda9*. A mutation in the dynein maturation factor gene *ODA8* shows SSNC with *wdr92*, *pf13*, and *pf23-1*. The *pf23-1* mutation shows SSNC with *pf13-1* and *wdr92* (Fig 6, inset lower left).

### The severity of the SSNC phenotype in dynein arm preassembly double heterozygotes is gene- and allele-specific

**Fig 7:**
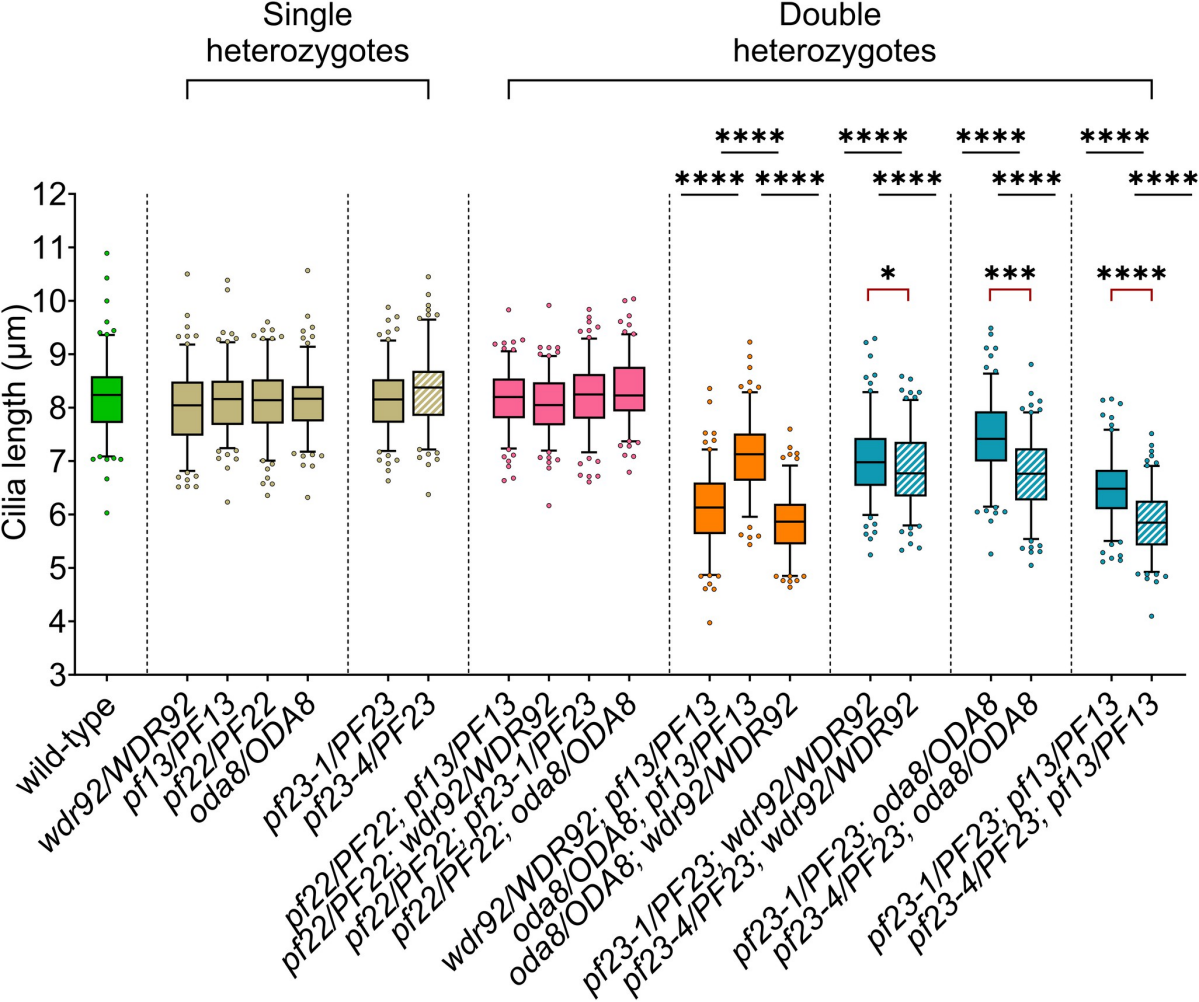
Cilia length of gametic diploids after regeneration for 180 min. Gametic diploids of the indicated genotypes were deciliated and allowed to regenerate for 180 min in 10 μg/ml cycloheximide. Three biological replicates of 50 individual cilia from 50 cells were collected for each strain. The cilia length of each strain is compared to the wild-type diploid (green). Striped fill indicates single or double heterozygotes generated using a null allele of *pf23* (*pf23-4*). Statistical analyses were performed using a one-way ANOVA with corrections for multiple comparisons. Red brackets highlight statistical comparison using a two-tailed t-test between double heterozygous diploids with either the *pf23-1* allele or *pf23-4* null allele. Circles above and below each box plot represent the 5^th^ and 95^th^ percentile respectively. ‘*’ p < 0.05; ‘**’ p < 0.001; ‘***’ p < 0.0001; ‘****’ p < 0.00001; ‘ns’ not significant.

In the SSNC screen, the strains were only allowed to assemble cilia for 40 minutes (Fig 6). Therefore, the cilia could be short because they had insufficient time to fully regenerate, or because the pool of proteins was exhausted. To address these two models, the cilia regeneration assay was repeated with a select group of single and double heterozygous strains for 180 min in the presence of cycloheximide (Fig 7, S11 Table). If the regeneration time was too short and the pool was not limiting, then we expect that after 180 mins (cilia grow to full-length after ~90 mins in wild-type) the cilia in these strains would resemble wild-type (Fig 4B). However, if the cytoplasmic pool is the limiting factor, the cilia of double heterozygotes that show SSNC should remain short even after 180 min since there are no more fully assembled complexes to contribute to cilia assembly. The wild-type diploids (Fig 7, green) assembled cilia 8.19 +/- 0.70 μm. *DNAAF* single heterozygous diploid strains all regrew cilia that were wild-type length (Fig 7, tan). As in the 40 min assay, the double heterozygotes with a *pf22* mutation regrew cilia with lengths comparable to wild-type (Fig 7, pink). Other double heterozygous diploids that include *oda8*, *wdr92*, and *pf13* show variable cilia assembly lengths that are shorter than wild-type (Fig 7, orange). This suggests that the cytoplasmic pool of proteins is altered in these strains, and this leads to the shorter cilia phenotype.

Because the *pf23-1* haploid mutant has a less severe phenotype than *pf23-4*, we asked whether the *pf23-4* allele exhibited a more severe SSNC phenotype in double heterozygous diploids than *pf23-1*. Double heterozygous diploids using the *pf23-4* allele in combination with the three mutations that showed SSNC with *pf23-1* (*wdr92*, *oda8*, *and pf13*) were generated and assayed for cilia length phenotypes. The double heterozygous diploids with the *pf23-4* allele (Fig 7, aqua, striped) regenerated shorter cilia than those with the *pf23-1* allele (Fig 7 aqua, solid). Since short cilia are associated with the loss of both ODAs and IDAs, and *pf23-1* can assemble more ODAs, this may explain the less severe phenotype [69].

### SSNC between *pf23* mutations and other preassembly/maturation factor mutations occurs in part due to dosage-dependent effects of *PF23*

**Fig 8:**
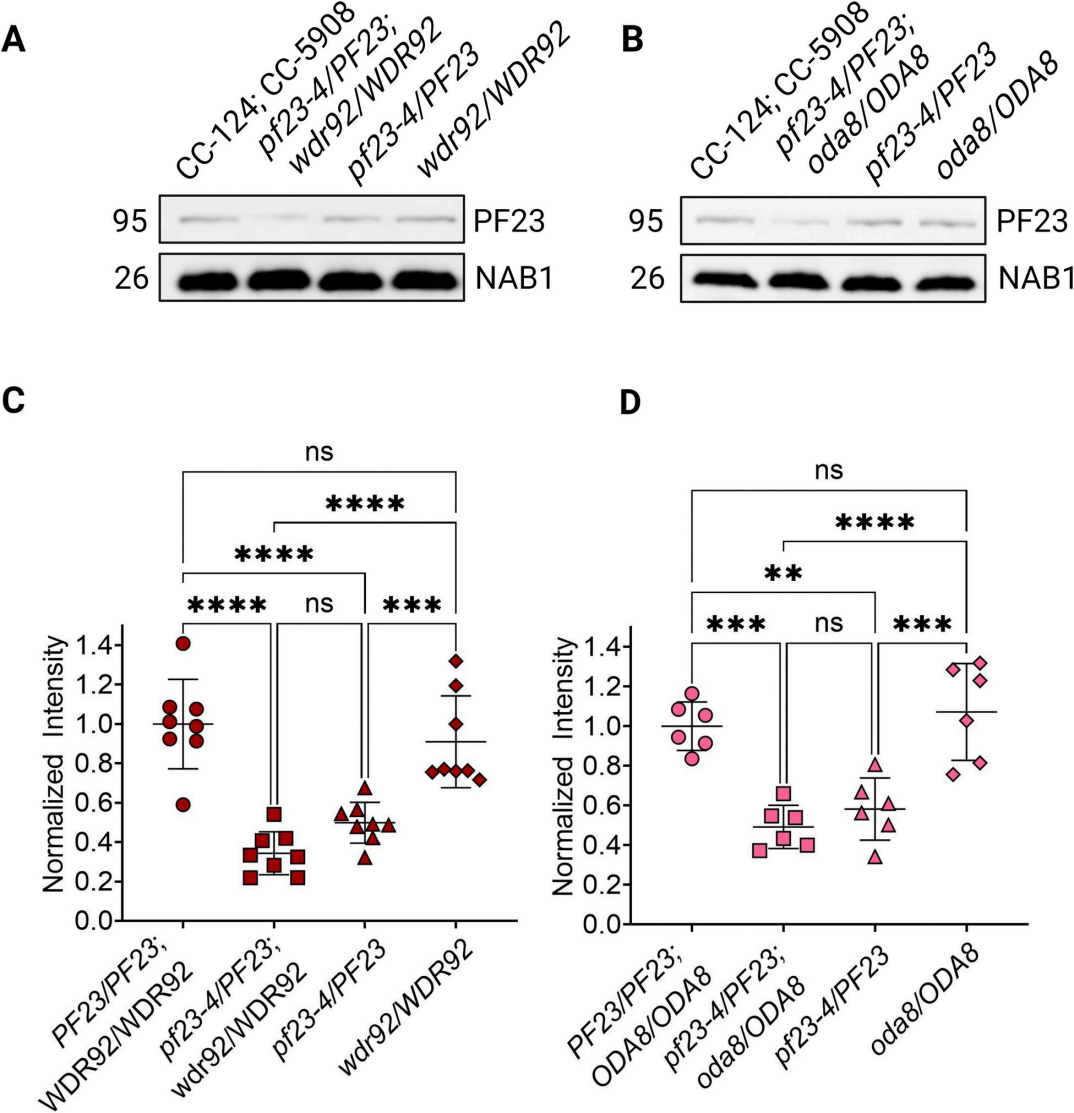
PF23 dynein arm preassembly factor protein is reduced by half in heterozygous strains at steady state. (A, B) Immunoblots of whole cell extracts of gametic diploid strains of the indicated genotypes. (C, D) Quantification of samples in A and B. A total of 50 μg of protein was loaded in each lane. Lane intensities were quantified and normalized to total protein stain. Samples were normalized to the mean of wild-type diploids (CC-124; CC-5908). Statistical analysis was performed using a one-way ANOVA with corrections for multiple comparisons. ‘*’ p < 0.05; ‘**’ p < 0.001; ‘***’ p < 0.0001; ‘****’ p < 0.00001; ‘ns’ not significant. Created with BioRender.

To further investigate the mechanism behind the SSNC phenotypes, we asked whether a reduction in the quantity of DNAAFs in the cytoplasm was altered and could be responsible for the SSNC phenotypes in the diploids. First, we examined the effect of gene dosage in steady-state gametic cells with the *pf23-4* null mutation in the absence of cycloheximide. If PF23 protein levels are allele dosage-dependent, *PF23* heterozygous diploids should have half wild-type levels of PF23. However, if there is upregulation of PF23 to compensate for the null allele, then PF23 levels should be closer to that of wild-type diploids. Immunoblots of whole cell extracts of wild-type diploids (CC-124/CC-5908), double heterozygote *pf23-4/PF23; wdr92/WDR92*, and single heterozygotes *pf23-4/PF23*, and *wdr92/WDR92* were analyzed (Fig 8, S12 Table). Approximately one-half of wild-type levels of PF23 are observed in the *pf23-4* single heterozygote, but wild-type levels of PF23 protein are produced in the *wdr92/WDR92* single heterozygote and the *oda8/ODA8* single heterozygote. Heterozygosity of these genes alone does not change the amount of PF23 protein. PF23 is not further reduced in *pf23* heterozygotes in the presence of heterozygosity for *wdr92* (Fig 8A, 8C) or *oda8* (Figs 8B, 8D, S7 and S14 Table). Therefore, the reduction of PF23 alone is not responsible for the decreased cilia length. The analysis of these double heterozygotes suggests that SSNC requires the additional reduction of either WDR92 or ODA8. Reduction in two preassembly proteins could result in a smaller cytoplasmic pool of mature ODAs and IDAs needed for cilia regeneration and be responsible for the short cilia phenotype.

**Fig 9:**
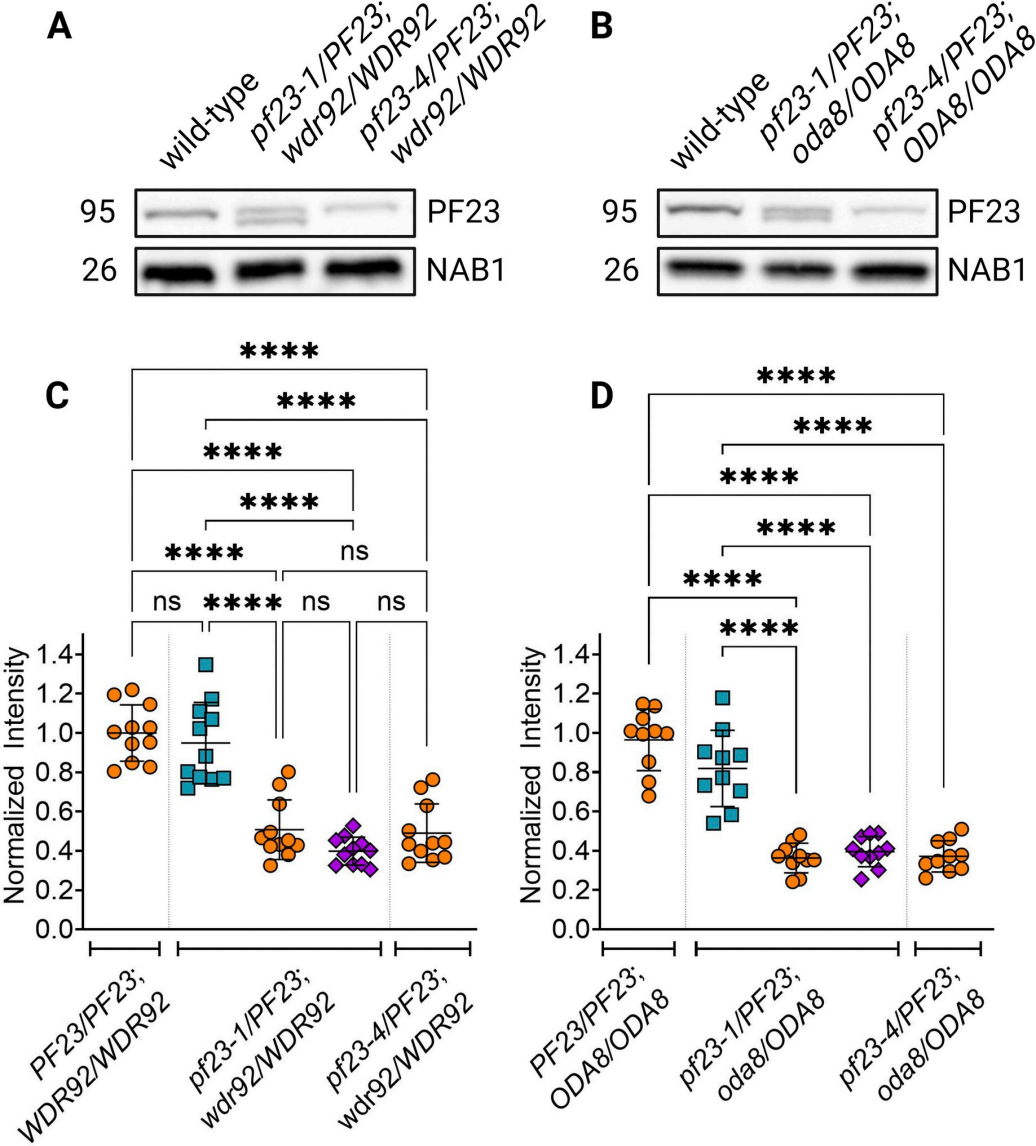
Cytoplasmic abundance of PF23 protein is dependent on the *pf23* allele present in heterozygous diploids. (A, B) Immunoblots of cytoplasmic extracts from gametic diploid strains that were deciliated and allowed to regenerate cilia for 60 min in 10 μg/ml cycloheximide. (C, D) Quantification of samples in A and B. A total of 50 μg of protein was loaded in each lane. Lane intensities were quantified and normalized to total protein stain. Samples were normalized to the mean of wild-type diploids (CC-124; CC-5908). Statistical analysis was performed using a one-way ANOVA with corrections for multiple comparisons. ‘*’ p < 0.05; ‘**’ p < 0.001; ‘***’ p < 0.0001; ‘****’ p < 0.00001; ‘ns’ not significant. Created with BioRender.

To further support the idea that the amount of PF23 is an important contributor to phenotype severity, we compared heterozygous diploids with the *pf23-1* and the *pf23-4* alleles in a heterozygous *wdr92* background (Fig 9, S13 Table). The heterozygous diploids were deciliated and allowed to assemble cilia in cycloheximide for 60 mins. Immunoblots of *pf23-1/PF23*; *wdr92/WDR92* and *pf23-4/PF23; wdr92/WDR92* diploids show that the *pf23-4/PF23; wdr92/WDR92* strain contained roughly half the quantity of PF23 compared to wild-type extracts. In the *pf23-1/PF23*; *wdr92/WDR92* diploid strain, each allele (*pf23-1* and *PF23*) contributes equal amounts of PF23 protein; the total was comparable to the wild-type diploid (Fig 9A, 9C). This was reproduced in the strains with double heterozygous mutations in *pf23* and *oda8* (Fig 9B, 9D). Together, the data from these two experiments show that PF23 is a dosage-dependent gene, and each allele contributes equally to PF23 protein in the cytoplasm without additional protein translation to compensate when there is loss of one allele. We conclude that reduced DNAAF dosage under the conditions tested in double heterozygotes is likely correlated with insufficient stable, pre-assembled dynein arms necessary to build cilia to the lengths observed in wild-type. This is supported by the data that shows when one-half the protein in the *pf23-1*/*PF23*; *wdr92/WDR92* strain is partially functional, in addition to the contribution from the wild-type copy, the cell’s ability to build longer cilia improves.

## Discussion

### The *pf23-1* deletion and *pf23-4* null alleles provide new insights into ODA and IDA preassembly in *Chlamydomonas*

The *pf23-1* and *pf23-4* strains show a major difference in assembly of outer dynein arms ([69], this work). We propose that the DYX domain, which is deleted in *pf23-1*, is responsible for IDA assembly. Since ODAs are reduced around 50% in the *pf23-1* strain, it is likely that the DYX domain and the remaining protein work cooperatively to properly assemble ODAs. Studies of *DNAAF4* mutations/variants in mice, zebrafish and humans that are presumed to be null show loss of both ODAs and IDAs from the cilia [40,43]. *Chlamydomonas* PF23 was suggested to only have a role in assembly of IDAs based on studies of the *pf23-1* allele. The isolation and characterization of a null *pf23* allele shows that PF23 is responsible for assembly of both ODAs and IDAs, as in other organisms. Equal amounts of wild-type and mutant protein are found in the *pf23-1/PF23* heterozygote. This indicates that while the DYX domain is important for PF23 function, it is not necessary for PF23 stability.

Previous studies showed that phosphorylation is important for IC138 function in cilia [25,88], and requires casein kinase 1 (CK1) [89]. We observed altered mobility of the IC138 protein in whole cell extracts of *pf23* mutants. Since IC138 is completely missing from isolated cilia based on our mass spectrometry and immunoblot data (Table 2, Fig 2), the difference in electrophoretic mobility must arise from modifications that occur in the cytoplasm. IC138 likely undergoes two independent modification event; one in the cytoplasm that is dependent on PF23, and another in the axoneme that requires CK1. We hypothesize that the cytoplasmic modification of IC138 is necessary for its assembly into the I1/f IDA dynein. An earlier study showed that IDA3, a proposed IDA adapter, must be present for IC138 to enter cilia, although a direct interaction was not tested [51]. Perhaps this cytoplasmic modification involving PF23 could facilitate IC138 transport into the axoneme through interactions with IDA3. Further experiments are needed to determine the nature of the modifications. It is not known if other preassembly factor mutants cause similar changes to IC138. Previous studies showed that ODAs require maturation factors (ODA5, ODA8, and ODA10) that help them assemble efficiently and make them competent for axonemal transport and/or binding. PF23 and other assembly factors may serve a similar role for IDAs.

### The cytoplasmic pool of axonemal proteins determines cilia regeneration capacity of haploids and diploids

*Chlamydomonas* haploid cells treated with cycloheximide contain a pool of proteins that is sufficient to assemble half-length cilia after deciliation [7]. We found that regenerated cilia of wild-type diploids are longer than haploids under these conditions; this is likely due to the difference of cytoplasmic pool sizes in haploid and diploid cells.

Having twice the genetic material of haploid cells is likely to lead to an increase in biosynthetic capacity in diploid cells [83]. In *Saccharomyces cerevisiae*, diploid cells are larger, and have double the number of ribosomes compared to haploid cells [90]. Although this has not been measured in *Chlamydomonas*, the same principle may apply. In addition to assembling longer cilia when protein synthesis is blocked, our results show that the rate of cilia regeneration in wild-type diploids is faster than in wild-type haploids. Since both the existing pool and *de novo* protein synthesis is required to assemble full-length cilia, we conclude that the faster rate of regeneration observed in these diploids must be a direct consequence of the larger pool size. Interestingly, diploid cells remain able to regulate the final ciliary length even when cilia assembly is faster than in haploids. Cilia length is tightly regulated in *Chlamydomonas* [91]. Changes in cilia length in mutants have not been associated with changes in cell size [88,92–95]. Analysis of diploid strains supports the idea that cilia length is independent of cell and cytoplasmic pool size.

The haploid motility mutants in our screen show a wide range of phenotypes (Fig 1). We anticipated that some double heterozygous diploids would show SSNC in steady-state cells with no stress. However, we observed no obvious motility phenotypes in any of the diploid strains. One explanation is that when the cells are grown under vegetative conditions for two days, there is enough time for them to synthesize the components necessary to build functional cilia. In our matrix, we tested 12 structural ciliary proteins. None of the mutants in these genes show cilia regeneration defects in diploids when protein synthesis is inhibited, which suggests that these structural proteins are not the limiting factor.

### The abundance and stability of cytoplasmic ODA and IDA proteins in *DNAAF* mutants influences cilia assembly

In the SSNC screen, cells must regenerate cilia using the cytoplasmic pool. In a wild-type diploid, we hypothesize that all the dynein proteins are stable. PF13, PF23, and WDR92, are DNAAFs that affect the stability of the dynein heavy chains. PF13 is necessary for both stability and assembly of ODAs, as the ODA heavy chains and one IDA subunit are reduced in the *pf13* mutant [68]. ODA and IDA subunits are reduced or absent in the cytoplasm of *pf23* and *wdr92* mutants, which shows a role in dynein stability [41,43,46,69]. In the diploids with double heterozygous mutations in two of these three DNAAFs, we hypothesize that the SSNC cilia phenotype occurs because there are insufficient preassembly complexes in addition to reduced dynein precursors available to build cilia.

The strains *pf13-1/PF13; oda6/ODA6*, and *pf13-1/PF13; oda9/ODA9* are heterozygous for mutations in a DNAAF and a dynein structural protein. IC2 is completely missing in the *oda6* mutant, and severely reduced in the *oda9* strain [76]. The IC1 and IC2 proteins stabilize each other [75]. We suggest that the SSNC in these two strains occurs due to a reduction in dynein proteins (IC1 and IC2) and reduced preassembly due to lower levels of PF13 in the cytoplasm.

In the *oda8* mutant cilia, only ODAs are lost. Wild-type levels of ODA subunits are found in the *oda8* cytoplasm. Like *pf13*, the *oda8* mutant fails to assemble ODA intermediate chains with ODA heavy chains, but ODA8 is not important for ODA subunit stability [47]. ODA8 has a role in maturing ODAs for transport into the cilia [48]. The SSNC we observe in ODA8 double heterozygotes could be due to the reduced production of mature dynein arms that can be transported into and bind the axoneme in combination with reduced dynein preassembly due to heterozygosity of a *DNAAF* in those SSNC pairs.

We did not detect SSNC in any double heterozygous diploids with *pf22* and another DNAAF. One explanation is that PF22 has a different role in dynein preassembly than other DNAAFs. In the *pf22* mutant cytoplasm, the ODA heavy chains are present at wild-type levels, but they fail to assemble. This suggests that while PF22 is important for ODA preassembly, it is not involved in ODA heavy chain stability [66]. PF22 does not interact with chaperone proteins as observed for other DNAAFs. In addition, recent studies show that PF22 structure resembles S-Adenosylmethionine-Dependent Methyltransferases. Mass spectrometry analysis shows that several ODA HCs, IDA HCs, IFT dynein and cytoplasmic dynein require PF22 for methylation of key residues [96].

### Dosage as a mechanism for SSNC in dynein arm preassembly factors can occur through reduction of multiple pathway components

In the *pf23-4/PF23*; *wdr92/WDR92* double heterozygote, PF23 is reduced by half based on our immunoblot data. We hypothesize that WDR92 is also reduced by half, although we were unable to test this for WDR92 and other DNAAFs in our SSNC screen due the lack of reagents. If WDR92 is also reduced by half in the *pf23-4/PF23*; *wdr92/*WDR92 strain, then ODA and IDA protein stability would be compromised and there would be fewer assembled dynein arms in the cytoplasm.

Having half the quantity of PF23 is enough for normal cilia regeneration since we do not observe phenotypes in *pf23/PF23* single heterozygotes. However, when this PF23 deficit is combined with a reduction in another preassembly factor, the effect of reduced preassembly machinery likely compromises the cell’s ability to produce enough dynein arms to rebuild the cilia. We were limited to testing SSNC among pre-existing mutant strains available through the *Chlamydomonas* Resource Center. Although there are currently 17 known DNAAFs, we were only able to obtain mutants in 5 of them; and except for *pf23-1*, they are all null alleles (Table 1). Tools for generating new alleles and tagged strains have been developed since this project was initiated. In the future, it will be interesting to test whether combinations of DNAAFs and chaperone proteins show SSNC.

### Can double heterozygosity in motile cilia genes play a role in human disease?

In humans, pathogenic variants in 58 genes [97] cause a rare disease called primary ciliary dyskinesia (PCD), characterized by recurrent lung infections, neonatal respiratory distress, bronchiectasis, otitis media, situs inversus/ambiguus, and male infertility [2,98,99]. Reports of SSNC or digenic inheritance (DI) with motile cilia genes are rare. One study identified patients with heterozygous variants in *DNAH6*, an IDA heavy chain gene, and another variant in *DNAI1*, an ODA intermediate chain gene. These individuals had heterotaxy, a PCD-associated phenotype. Knockdown of *DNAH6* in mice heterozygous for *dnaI1* resulted in motile cilia defects, which suggests that heterozygous *dnah6* and *dnai1* mutations are responsible for the patient phenotypes [100]. Roughly 30% of patients with a clinical PCD diagnosis have unknown genetic etiology despite genetic testing and/or exon sequencing. A small subset of patients with a PCD diagnosis are heterozygous for variants in one or more PCD genes [101–104]. This implies that either the correct causative gene was missed due to a complex variant, a variant in a PCD gene was not included in the test set, a variant is in a non-coding region, or the second hit is in a different gene than the one already identified [103–107]. Our results reinforce the findings that PCD mutant alleles show a recessive inheritance pattern. Although our steady-state double heterozygous diploids do not show obvious motility or cilia defects, the possibility of SSNC should be considered when looking at human PCD patients. Individuals with milder PCD phenotypes might be candidates for SSNC. Double heterozygosity phenotypes may be exacerbated under stress conditions that include viral infections and other environmental exposures and produce PCD-like phenotypes. The implications of this work are that individuals who are double heterozygotes for two motile cilia genes may encounter difficulties with recovering from assaults that damage their airways.

## Materials and methods

### Strains and diploid generation here

The strains used in this study are listed in Table 1. To generate diploid strains, single mutant haploid strains with the *AC17* allele were crossed with strains CC-5908 or CC-5909 with the genotype *ac17; aphviii-TG* (S1 Table) that are mating-type plus or mating-type minus, respectively. Mutant strains with reciprocal selectable genotypes were mated and then plated onto acetate-free plates supplemented with 10 μg/ml paromomycin sulfate (Sigma Aldrich, P-8692, lot #101K1943). Plates were incubated in constant light at 25°C for 3–5 days until colonies appeared. Each diploid colony was screened by PCR for heterozygosity at the mating-type locus before further analysis. Available PCR markers were used to genotype strains when appropriate. Primers are listed in S1 Table. Strain CC-5485 with *aphviii* insertions was used to obtain strains CC-5908 and CC-5909. The *aphviii* insertion in CC-5485 does not cause a motility phenotype [85]. The *oda11* strain CC-2672 from the *Chlamydomonas* Resource Center has a second hit that results in short cilia. This strain was backcrossed to wild-type to remove it.

### Deciliation, regeneration, and imaging

For deciliation and regeneration assays, cells were plated onto R (rich) medium plates for two days at 25°C [108,109]. They were transferred into 1 ml of liquid R medium in a 1.5 ml Eppendorf tube and rocked under light for 3 hrs to allow cilia to assemble. The cells were placed on ice, spun at 10,000 x g for 30 sec and resuspended in 1 ml cold deciliation solution (10 mM Tris pH 7.5 (Fisher Scientific, BP152-S, lot #190374), 150 mM D-mannitol (Merck, 654833, lot #K95690782-930) and 1mM CaCl_2_.2H_2_O (Mallinckrodt Specialty Chemicals, 4160, lot #4160-KLBY)). pH shock was performed with 30 μl 0.5N acetic acid (Sigma Aldrich, 695092–2.5L, lot #4102327022) followed by 30 μl 0.5M sodium hydroxide (STREM Chemicals Inc., 93–1063, lot #35205J00) [51] and then checked for deciliation by phase microscopy with a 40x objective. Cells were centrifuged and then resuspended in 500 μl R medium with or without 10 μg/ml cycloheximide (Sigma Aldrich, C-6255, lot #1793) and allowed to regenerate cilia for the times indicated. A 40 min timepoint was selected for most experiments, unless indicated otherwise, because it allowed screening to be completed in a reasonable amount of time. Cells were fixed with a 1:10 dilution of 2% glutaraldehyde (Sigma Aldrich, G5882-100ML, lot #056K5318) in 0.2 M phosphate buffer pH 7.5 (0.31 g NaH_2_PO4 (Honeywell, 04269-1KG, lot #10185639)) and 1.09 g Na_2_HPO4 (Fisher Scientific, S25563A, lot #0GK20031204A) in 50 ml water. Cells were then allowed to adhere to glass slides coated with poly-L-Lysine solution (Sigma Aldrich, P8920-500ML, lot #SLCM0307). Excess cell volume was aspirated, and the slides were air-dried before imaging. For initial diploid screening of cycloheximide treated cells, fixed samples were imaged using phase contrast microscopy on a Zeiss AxioPhot with a 20x Neofluar objective lens and a NA of 2.0 (Carl Zeiss AG, Oberkochen, Germany). Images were captured using a Phantom Miro eX2 camera and Phantom Camera Control Application 2.6 (Vision Research, Wayne, NJ) using 640 x 480 resolution. Cilia length was quantified using ImageJ. For immunofluorescence staining, a previously described protocol was used [79]. The primary antibody used was anti-acetylated α-tubulin mouse monoclonal clone 6-11B-1 (1:500 dilution, Sigma, T7451-200, lot #077M4751V). The secondary antibody was Alexa Fluor 488 donkey anti-mouse IgG (1:1,000 dilution, Invitrogen, A21202, lot #2266877). Images were false color stained using ImageJ when appropriate.

### Genome editing

CRISPR genome editing protocol and associated reagents are described [79].

### Axoneme isolation and mass spectrometry

Axonemes of wild-type strain 4-2P (generated from a cross between CC-124 and CC-125), strains *pf23-4; cnk11-3* (2–3, 3–1 and 5–1), and *cnk11-3* (1–7, 1–4, and 5–1) [110] were prepared as sets of three biological replicates using the dibucaine method [111]. One hundred μg of protein from each sample were submitted for tandem mass spectrometry analysis to the Danforth Plant Science Center St. Louis, MO [112].

### Immunoblotting

Immunoblotting was performed using gametic whole cell extracts. Protein from the cytoplasm and the cilia are included in the extract where indicated. Each strain was grown on one TAP [113] plate for 2 days at 25°C, then placed at room temperature for 3 days to allow cells to become gametic. Each plate of cells was resuspended in 10 ml of M-N/5 (minimal minus nitrogen / 5) [109] and rocked in light at room temperature for 3 hrs. Cells were spun at 2,000 x g for 5 min, then resuspended in 10 ml of autolysin [114] for 1 hr. Next, cells were spun at 2,000 x g for 5 min and resuspended in 750 μl of cell lysis buffer (20 mM Tris-HCl pH 7.5, 5 mM MgCl_2_ (Sigma, 232–094, lot #088J00292), 300 mM NaCl (Fisher Scientific, BP358-212, lot #976181), 5 mM Dithiothreitol (RPI Research Products, D11000-5.0, lot #12936482), 0.1% Triton-X100 (EM Science, TX1566-1, lot #31050) [64], with 20 μM phenylmethylsulfonyl fluoride (Sigma, P-7626, lot #050M1688) and the Protease Inhibitor Cocktail for plants (Sigma, p9599, lot #086K4075). The cell suspension was passed 10 times through a 22 ½ gauge needle and syringe, or frozen overnight at -80°C to generate cell lysate. The lysate was spun at 21,000 x g for 30 min to remove non-lysed cell bodies, organelles and debris. The supernatant was transferred to a fresh 1.5 ml Eppendorf tube and protein concentration was measured using the Bradford assay (Bio-Rad Protein Assay Dye Reagent Concentrate (#5000006, lot #64227541). Samples were frozen at -80°C. For protein electrophoresis, 5x sample buffer (3.78 g Tris, 5 g SDS (sodium dodecyl sulfate, MP Biomedicals, 811030, lot #M3313), 25 g sucrose (EMD Chemicals Inc., SX1075-3, lot #21H1956938), in 90 ml, pH to 6.8, 0.04 g bromophenol blue (Sigma Aldrich, 1PVH00010, lot #114391-5G) in a final volume of 100 ml) with freshly added β-mercaptoethanol (Fisher Scientific, BP176-100, lot #990569) to a final concentration of 5% was added to the sample at 1X concentration and heated in a dry bath for 10 min at 70°C. Fifty μg of each sample was loaded onto a 4–20% Novex Tris-Glycine gel (XP04205BOX) and run at 225 volts for 45 min. Following transfer onto Immobilon-P 0.45μm PVDF membranes (Sigma Aldrich, 1PVH00010, lot #0000224124), each membrane was incubated with Invitrogen No-Stain Protein Labeling Reagent (Invitrogen, A44449, lot #2656135) according to manufacturer’s instructions. Subsequent blotting steps followed standard procedures [62]. For antibody probing, the membranes were cut horizontally at the appropriate size marker for the protein to be examined. Each section of membrane was incubated with its respective primary and secondary antibody sequentially. Primary antibodies used were rabbit anti-PF23 CT299 [46] (1:1000, a gift from Dr. Stephen King), rabbit anti-IC138 (1:2000, a gift from Dr. Winfield Sale and Dr. Lea Alford), rabbit anti-RIB43a and DRC2 (1:2000, a gift from Dr. Mary Porter), rabbit anti-NAB1 (1:10,000, Agrisera, catalog #AS08 333, lot #1311), and monoclonal mouse anti-IC2 clone 1869A (1:10,000, Sigma, D-6168, lot #92H4862). Secondary antibodies used were goat anti-rabbit HRP (Sigma-Aldrich, A6154-1ML, lot #SLCL2476) at 1:5000, 1:5000, 1:10,000, and 1:10,000 dilutions respectively. Goat anti-mouse HRP (catalog 82–8520, lot #WD321502) was used at 1:10,000 dilution. Membranes were visualized using the Invitrogen iBright 1500 Imager with the universal channel to detect total protein stain and chemiluminescence.

### Recombineering and rescue of *pf13-1* and *pf23-1*

To rescue the *pf13-1* strain, we utilized the recombineering approach detailed in Emrich-Mills *et al*. [64]. The BAC strain 23P6 containing the *PF13* gene (Cre09.g411450, Phytozome v5.6) was obtained from the *Chlamydomonas* BAC library (http://chlamycollection.org) [115]. Recombineering construct PLM099 (S2A Fig) that contains the Venus-YFP-FLAG tag and *aphviii* paromomycin resistance cassette was chosen for recombineering with 23P6. Following recombineering, two colonies were selected for further analysis after running isolated plasmid DNA on a 0.8% agarose gel and checking for the expected size (S2B Fig). The two colonies were then digested with restriction enzymes *Nde*I and I-*Sce*I to confirm the presence of the PLM099 backbone and the *PF13* gene (S2C Fig). DNA from the *PF13*:*YFP-FLAG* #2 plasmid was isolated from a 4 ml overnight culture and used to transform *pf13-1* as described [79]. PCR to detect the presence of the transgene was performed on a subset of colonies (S2D Fig) that showed a rescued phenotype of swimming (*pf13-1* is non-motile) in liquid 96-well plates under a dissecting microscope. To identify the transgene, PCR primers were used to amplify the junction between the 3’ end of *PF13* and the 5’ end of the YFP-FLAG tag (S2E Fig, S1 Table). The presence of the tagged protein was identified by immunoblotting using anti-FLAG antibody (F1804 monoclonal antibody, clone M2 in mouse, lot #SLCK5688, Sigma Aldrich, S2F Fig). Rescue was confirmed by measuring cilia length, swimming velocity, and beat frequency (S2G–S2I Fig). Similar steps were followed for rescue of *pf23-1*.

### RNA and cDNA isolation and analysis

Cells for RNA isolation were collected from R plates grown in bright light for 2 days at 25°C. RNA was prepared using Trisol Reagent (Ambion, 15596018, lot #265712) and quality was assessed using a 0.8% agarose gel. cDNA was generated using SuperScript IV VILO MasterMix (Invitrogen, 11756060, lot #00837183) [112]. Primers in S1 Table were used to detect cDNA.

### Whole genome resequencing and analysis

Whole genome sequencing was performed as described previously [62]. In instances where SnpEff [63, 116] was unable to identify the causative mutation, the whole genome resequencing data was manually analyzed using IGV and alignment to reference sequences CC-124 and CC-125.

### Graphing, Statistical Analysis and Image generation

Generation of graphs and statistical analyses were performed using GraphPad Prism 10.

## Supporting information

S1 Fig Whole genome resequencing analysis of the *pf13-1* and *oda4-1* strains. (A) Reads spanning the *PF13* locus (Cre09.g411450) from the *pf13-1* mutant show a 44.7 kb deletion in the mutant strain compared to wild-type CC-124 and CC-125 strains. Brackets with numbers indicate the location of the 7 genes deleted in *pf13-1*. (B) The genes and corresponding protein products deleted in the *pf13-1* strain as annotated in Phytozome 13 [118]. Numbers indicate the order in which the genes are arranged as in (A), starting with *PF13*. Arrows indicate PCR confirmation of the regions deleted in *pf13-1* using primers listed in S1 Table. The green check marks show that the sequence is amplified in both wild-type (CC-124) and *pf13-1* and indicate the regions just outside the deletion breakpoints on either side. A red ‘X’ indicates that the sequence between the primer pair was amplified in wild-type but not in *pf13-1*. (C) IGV snapshot of reads spanning the region of the *oda4-1* (Cre09.g403800) showing a 5 bp deletion in exon 22 out of 30 exons. (A, C) The region containing the deletions is indicated with a red arrow and bracket. Created with BioRender.
(TIF)

S2 Fig The ciliary assembly defect of *pf13-1* is rescued by transformation with a *PF13*:*YFP-FLAG-TG* construct generated using recombineering cloning. (A) Amplification of the PLM099 recombineering plasmid that contains the YFP-FLAG tag and paromomycin selectable marker with PF13-specific 50 bp homology primers (S1 Table). (B) DNA isolated from two independent bacterial colonies with PLM099 after recombineering with BAC 23P6 (from the *Chlamydomonas* BAC library) containing *PF13*. The top band (labeled with an asterisk) likely represents BAC DNA that did not undergo recombineering. (C) Restriction digest of DNA from the two colonies isolated in (B). I-*Sce*I is used for linearization of the plasmid, while *Nde*I + *Xba*I digest indicates the presence of the PLM099 vector backbone along with the *PF13* gene. (D) PCR was performed on a subset of *Chlamydomonas pf13-1* strains transformed with *PF13*:*YFP-FLAG* #2 to verify the presence of YFP-FLAG tag using primers within the *Venus-YFP* sequence. A strain with *CAH6*:*YFP-TG* [119] was used as a positive control. (E) PCR confirmation of fusion of the *YFP-FLAG* tag with the 3’ end of the *PF13* gene in two of the strains (A2 & B8 renamed *pf13-1; PF13*:*YFP-FLAG-TG* #1 and #2 respectively) tested in (D). (F) Immunoblot of transformed strains in (E) using an anti-FLAG antibody. The expected size of PF13 is 75 kDa. The observed bands are larger due to the YFP and 3x FLAG epitope tags present at the C-terminus of PF13. (G-I) Cilia length, swimming velocity, and beat frequency are rescued in the strains *pf13-1; PF13*:*YFP-FLAG-TG* #1 and #2. CC-124 and CC-125 are wild-type controls. *pf13 is* non-motile and indicated by an ‘X’. Created with BioRender.
(TIF)

S3 Fig Isolation and analysis of wild-type strains transformed with *aphvii* targeted to exon 1 of the *PF23* gene. (A) PCR screening of 7 transformants from *PF23* CRISPR insertional mutagenesis. One colony, B10 fails to amplify the exon 1 region targeted by the primers and amplifies the *aphvii-PF23* junction in exon 1. (B) Strain H9 was retrieved by screening an additional 11 strains from the same transformation. The insertion is oriented in the forward direction. (C) A second transformation produced strains 2–3 (*pf23-2)* and 4–3 (*pf23-3*). The insertion in both strains is oriented in the reverse direction. (D) cDNA analysis of *PF23* CRISPR transformants to detect whether mRNA is disrupted. A band was amplified in strain B10, and it was excluded from further analysis. Strains 2–3, 4–3 and H9 (*pf23-2*, *pf23-3*, and *pf23-4* respectively) failed to generate a cDNA amplicon for exons 1 and 2. Created with BioRender.
(TIF)

S4 Fig Genotyping of *pf23-1; PF23* transgenic rescue strains. Strain *pf23-1* contains an in-frame deletion in exon 5 and was rescued with wild-type *PF23* constructs tagged with either m-scarlet or neon green. These are listed at the top of Fig S4. The labels on the right indicate the primers used (See S1 Table). (A) The CC-124 and *pf23-4* strains both amplify wild-type PCR products at exon 5. The *pf23-1* amplicon is smaller due to the in-frame deletion. Both rescue strains carry the smaller *pf23-1* amplicon and a wild-type exon 5 amplicon. (B) Amplification of the junction between the 3’ end of *PF23* and the 5’ end of m-scarlet in the rescue strain. Asterisks indicate non-specific bands. (C) Amplification of the junction between the 3’ end of *PF23* and the 5’ end of neon green. (D) Amplification of the junction between the 3’ end of *PF23* and *aphvii* in the *pf23-4* null strain with an insertion in exon 1. (E) *pf23-4* fails to amplify a band in exon 1 due to the *aphvii* insertion. Created with BioRender.
(TIF)

S5 Fig Genotyping of diploid strains using PCR markers against selectable markers for *ac17* and *aphviii*. Haploid strains CC-5908 and CC-5909 carry an insertion in the *ATG17* gene and a mutation in *ac17* but are wild-type swimmers. CC-124 and CC-125 are wild-type at both loci. The three diploid strains are heterozygous for the *atg17* and *ac17* mutations. (A) PCR primers for the *ATG17* gene [85] were used to detect the wild-type copy of the gene in CC-124, CC-125, and three diploid wild-type strains (lanes 3A-7A). No amplification is observed in CC-5908 and CC-5909 (lanes 1 & 2), since the *aphviii* paromomycin resistance gene is inserted into the *atg17* gene and is too large to amplify with the condition used. (B) PCR primers were designed to detect the junction between the *ATG17* gene and the *aphviii* insertion in the *ATG17* gene. This junction is present in the CC-5908 and CC-5909 strains that carry the insertion (lanes 1B-2B), as well as the heterozygous diploids (lanes 5B-7B). (C) PCR primers were designed to amplify across the region containing the mutation in the *AC17* gene [120]. Digestion with *Hae*III generates different sized products in the *ac17* containing strains (lanes 1C-2C) compared to CC-124 and CC-125 wild-types (lanes 3C-4C). The heterozygous diploid strains (5C-7C) generate both sets of digest products. (D) A map of the *AC17* PCR product digested with *Hae*III. Created with BioRender.
(TIF)

S6 Fig SSNC between double heterozygous mutants in IFT in *Chlamydomonas* diploid strains. The *ift* mutants shown are temperature-sensitive or nonconditional (indicated by *). At the permissive temperature (21°C), these mutant strains assemble cilia and swim. After 12 hrs at the restrictive temperature (32°C), the strains are aciliate. The mutations are recessive; single heterozygotes assemble cilia at the restrictive temperature (left column). Several members of the same complex fail to complement as double heterozygotes (red squares represent IFTA, blue squares are IFTB). Interestingly, *ift172* complements other components of the IFTB complex but fails to complement mutants of IFTA (yellow squares). The data were generated using methods described in Iomini *et al*. The original published SSNC combination of mutants *ift144* and *ift139* is highlighted by a pink star [82]. Created with BioRender.
(TIF)

S7 Fig Quantification of PF23 abundance in diploid and haploid strains with different *WDR92* genotypes. (A) Representative blot with 50 μg protein of the indicated strains. Each strain within the brackets indicates a biological replicate. Haploid strains with genotype wild-type *WDR92* included CC-5908 and CC-124. IC2 and NAB1 are used as loading controls. Total protein stain was used for quantification. (B) Quantification of samples (biological and technical replicates). All samples were normalized to the mean of the wild-type diploids. A one-way ANOVA was used to assess statistical significance. ns: not significant. Created with BioRender.
(TIF)

S1 Table PCR primers generated to genotype strains used in this work. Where indicated, the PCR product is digested with a restriction enzyme to distinguish between wild-type and mutant genotypes. ‘No band in mutant’ indicates the presence of an insertion that is too large to amplify during the extension time needed to amplify the PCR products with the same primers in wild-type. *Designed using the method for allele-specific primers (ASP) [117]. ** The mating-type primers can be run simultaneously to generate two bands that distinguish between both mating-types.
(DOCX)

S2 Table The proteins characterized in wild-type, the *pf23-4; cnk11*, and *cnk11* mutants by mass spectroscopy. The protein, gene, and mutant names are in columns 1–3. The normalized log_2_ values from the average of three biological replicates are presented. Proteins in the MIA complex, radial spokes, N-DRC, Central apparatus, and MIPs are presented to show small or no changes.
(DOCX)

S3 Table SSNC observed for IFT mutants. Each mutation is classified by the IFT complex in which it has been found. The human ortholog of each *Chlamydomonas* IFT gene is listed. **These strains produce a mutant protein with partial function at the permissive temperature. ^#^Null at the restrictive temperature.
(DOCX)

S4 Table Cilia length, swimming velocity, and beat frequency of *Chlamydomonas* haploid mutants. (XLSX)

S5 Table SNPEff variant output from whole genome sequencing analysis of haploid mutants. (XLSX)

S6 Table Cilia lengths of *pf23* mutants and *pf23* rescue strains. (XLSX)

S7 Table Comparison of cilia length, cell body length, swimming velocity, and beat frequency of *Chlamydomonas* haploids and diploids. (XLSX)

S8 Table Swimming velocity, beat frequency, and cilia length of *Chlamydomonas* single and double heterozygous diploids. (XLSX)

S9 Table Cilia length of regenerating haploids and diploids +/- cycloheximide. (XLSX)

S10 Table Cilia length of regenerating single and double heterozygous diploids 40 mins post-deciliation in cycloheximide. (XLSX)

S11 Table Cilia lengths of single and double heterozygous diploids 180 mins post-deciliation in cycloheximide. (XLSX)

S12 Table Normalized immunoblot intensities of PF23 abundance in gametic whole cell extracts from double heterozygous diploids with *pf23-1* and/or *wdr92* or *oda8* mutations. (XLSX)

S13 Table Normalized immunoblot intensities of gametic cytoplasmic extracts of PF23 abundance in diploids heterozygous for *pf23-1* or *pf23-4* and *wdr92*. (XLSX)

S14 Table Normalized immunoblot intensities of PF23 abundance in gametic haploid and diploid strains with *wdr92* mutations. (XLSX)
