# Supplementary figures and images for: Gene dosage of independent dynein arm motor preassembly factors influences cilia assembly in Chlamydomonas reinhardtii
Source: PLoS Genet. 2024 Mar 18;20(3):e1011038. doi: 10.1371/journal.pgen.1011038 (PMC11020789; doi:10.1371/journal.pgen.1011038)

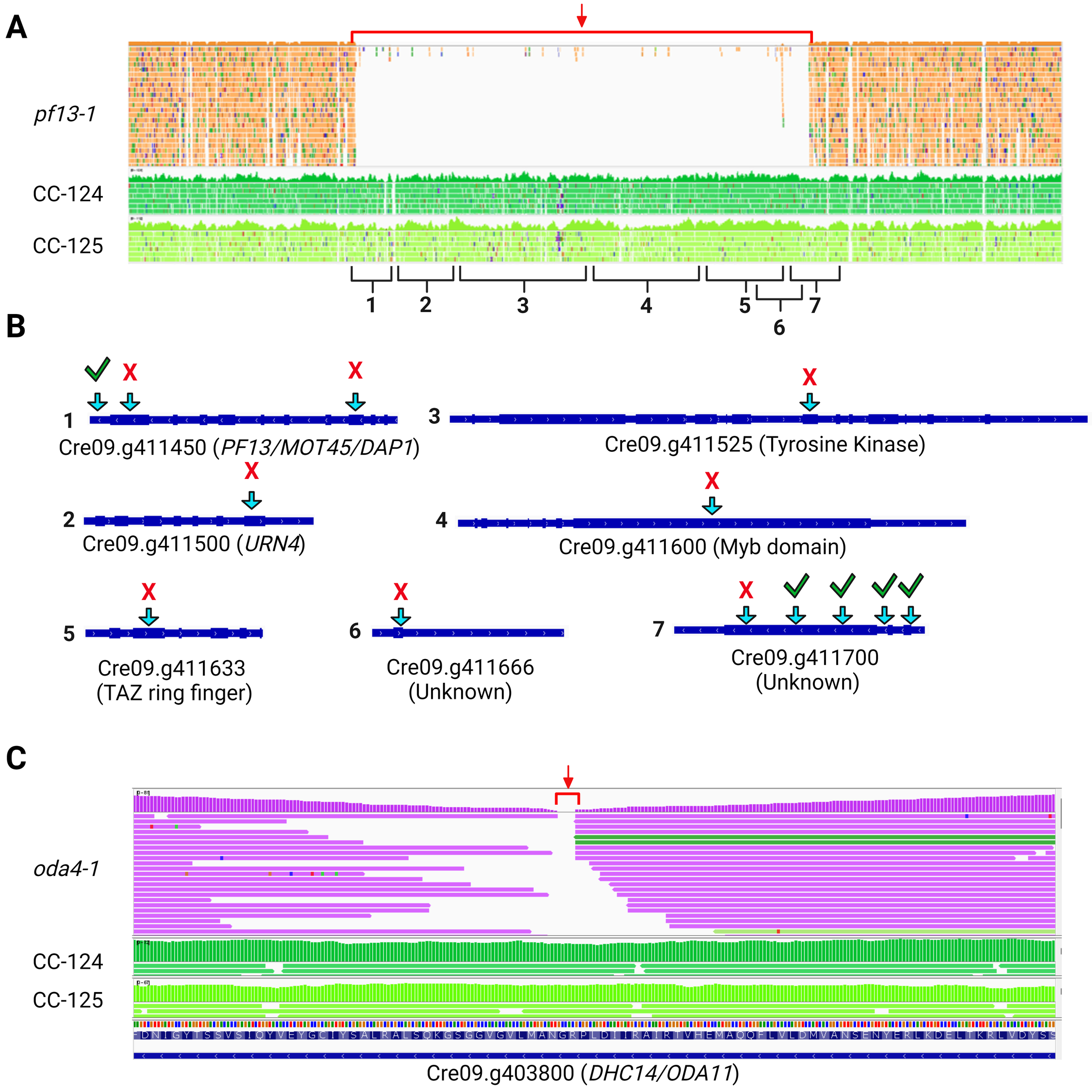

Supplement: S1 Fig — (A) Reads spanning the PF13 locus (Cre09.g411450) from the pf13-1 mutant show a 44.7 kb deletion in the mutant strain compared to wild-type CC-124 and CC-125 strains. Brackets with numbers indicate the location of the 7 genes deleted in pf13-1. (B) The genes and corresponding protein products deleted in the pf13-1 strain as annotated in Phytozome 13 [118]. Numbers indicate the order in which the genes are arranged as in (A), starting with PF13. Arrows indicate PCR confirmation of the regions deleted in pf13-1 using primers listed in S1 Table. The green check marks show that the sequence is amplified in both wild-type (CC-124) and pf13-1 and indicate the regions just outside the deletion breakpoints on either side. A red ‘X’ indicates that the sequence between the primer pair was amplified in wild-type but not in pf13-1. (C) IGV snapshot of reads spanning the region of the oda4-1 (Cre09.g403800) showing a 5 bp deletion in exon 22 out of 30 exons. (A, C) The region containing the deletions is indicated with a red arrow and bracket. Created with BioRender. (TIF) [file pgen.1011038.s001.tif]

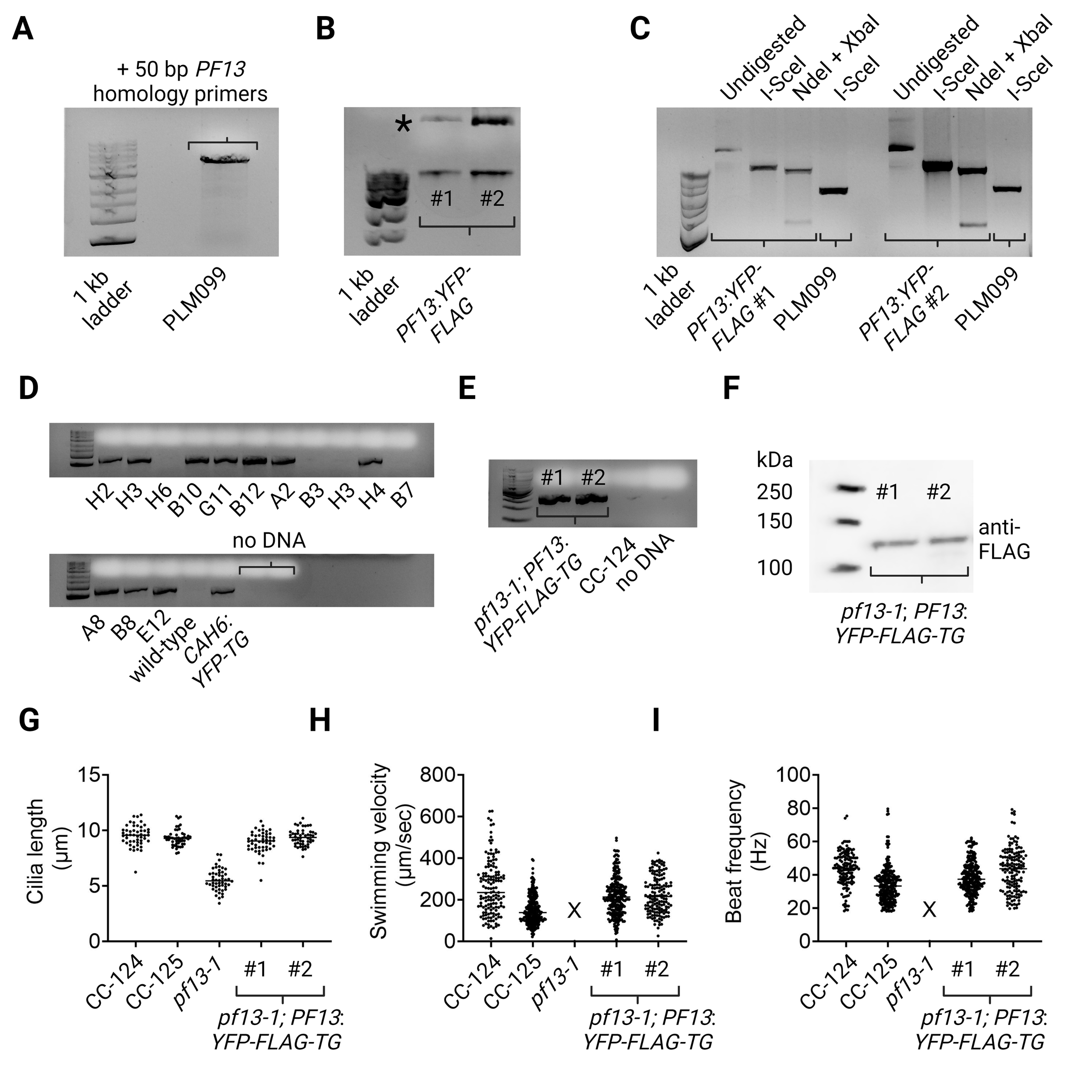

Supplement: S2 Fig — (A) Amplification of the PLM099 recombineering plasmid that contains the YFP-FLAG tag and paromomycin selectable marker with PF13-specific 50 bp homology primers (S1 Table). (B) DNA isolated from two independent bacterial colonies with PLM099 after recombineering with BAC 23P6 (from the Chlamydomonas BAC library) containing PF13. The top band (labeled with an asterisk) likely represents BAC DNA that did not undergo recombineering. (C) Restriction digest of DNA from the two colonies isolated in (B). I-SceI is used for linearization of the plasmid, while NdeI + XbaI digest indicates the presence of the PLM099 vector backbone along with the PF13 gene. (D) PCR was performed on a subset of Chlamydomonas pf13-1 strains transformed with PF13:YFP-FLAG #2 to verify the presence of YFP-FLAG tag using primers within the Venus-YFP sequence. A strain with CAH6:YFP-TG [119] was used as a positive control. (E) PCR confirmation of fusion of the YFP-FLAG tag with the 3’ end of the PF13 gene in two of the strains (A2 & B8 renamed pf13-1; PF13:YFP-FLAG-TG #1 and #2 respectively) tested in (D). (F) Immunoblot of transformed strains in (E) using an anti-FLAG antibody. The expected size of PF13 is 75 kDa. The observed bands are larger due to the YFP and 3x FLAG epitope tags present at the C-terminus of PF13. (G-I) Cilia length, swimming velocity, and beat frequency are rescued in the strains pf13-1; PF13:YFP-FLAG-TG #1 and #2. CC-124 and CC-125 are wild-type controls. pf13 is non-motile and indicated by an ‘X’. Created with BioRender. (TIF) [file pgen.1011038.s002.tif]

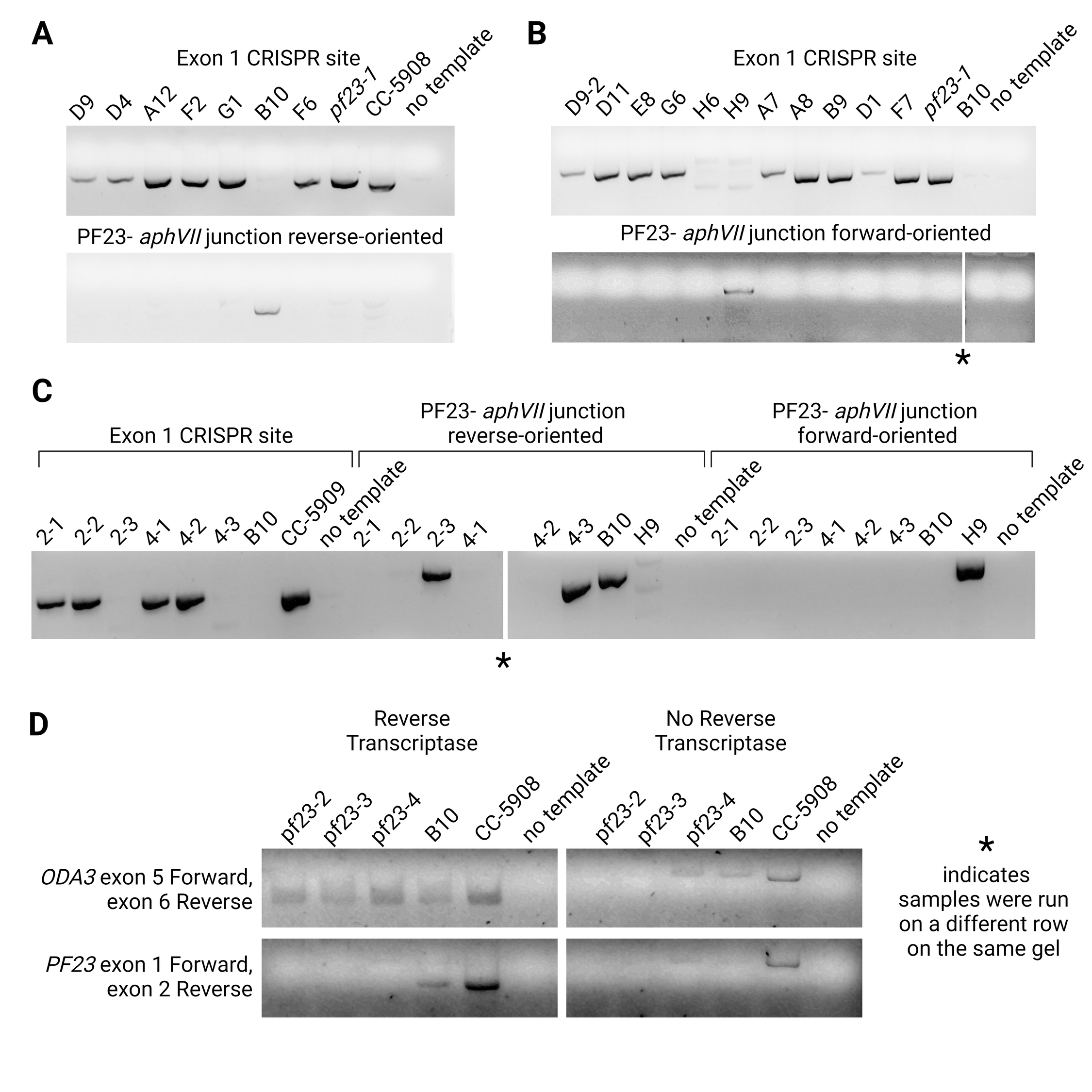

Supplement: S3 Fig — (A) PCR screening of 7 transformants from PF23 CRISPR insertional mutagenesis. One colony, B10 fails to amplify the exon 1 region targeted by the primers and amplifies the aphvii-PF23 junction in exon 1. (B) Strain H9 was retrieved by screening an additional 11 strains from the same transformation. The insertion is oriented in the forward direction. (C) A second transformation produced strains 2–3 (pf23-2) and 4–3 (pf23-3). The insertion in both strains is oriented in the reverse direction. (D) cDNA analysis of PF23 CRISPR transformants to detect whether mRNA is disrupted. A band was amplified in strain B10, and it was excluded from further analysis. Strains 2–3, 4–3 and H9 (pf23-2, pf23-3, and pf23-4 respectively) failed to generate a cDNA amplicon for exons 1 and 2. Created with BioRender. (TIF) [file pgen.1011038.s003.tif]

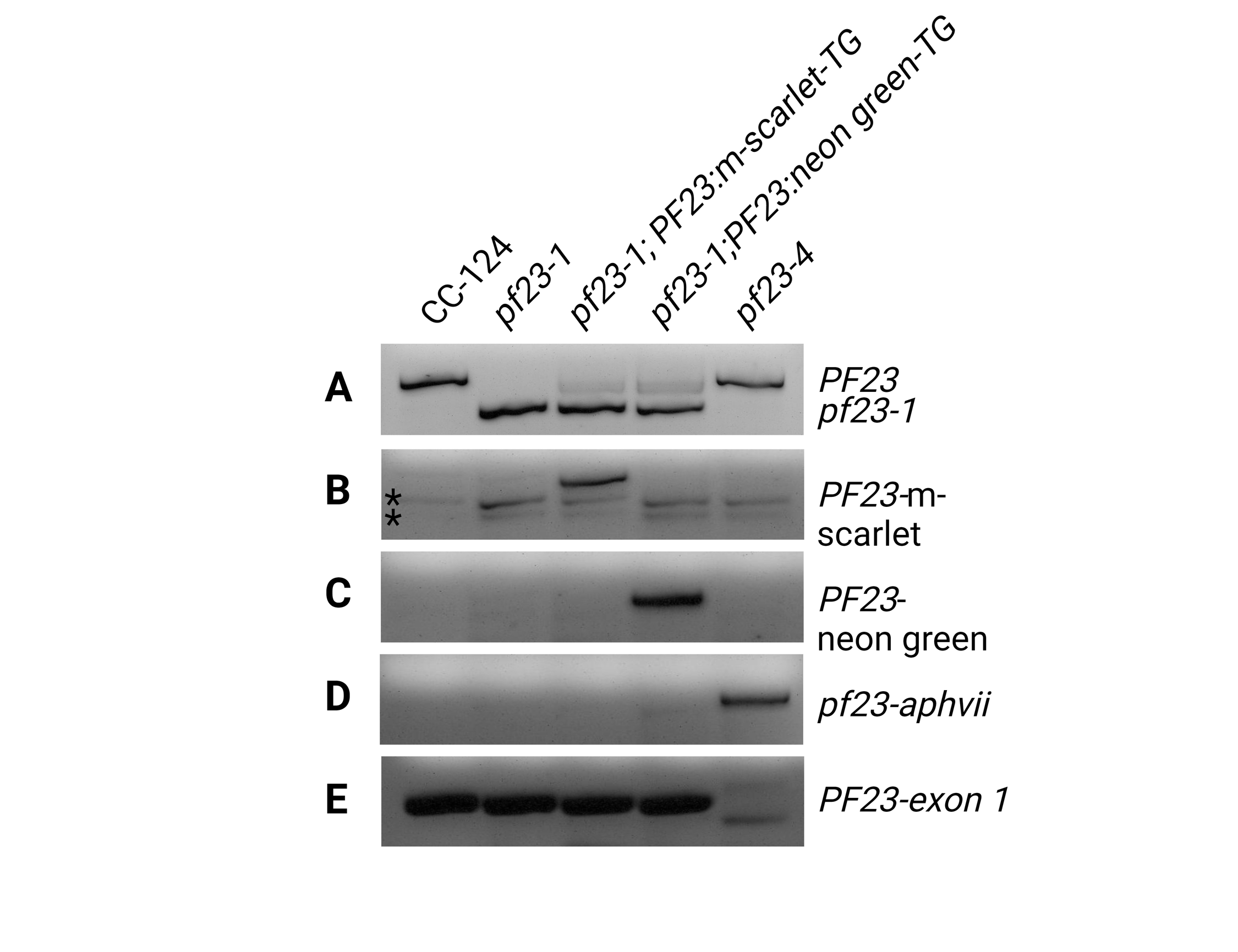

Supplement: S4 Fig — Strain pf23-1 contains an in-frame deletion in exon 5 and was rescued with wild-type PF23 constructs tagged with either m-scarlet or neon green. These are listed at the top of Fig S4. The labels on the right indicate the primers used (See S1 Table). (A) The CC-124 and pf23-4 strains both amplify wild-type PCR products at exon 5. The pf23-1 amplicon is smaller due to the in-frame deletion. Both rescue strains carry the smaller pf23-1 amplicon and a wild-type exon 5 amplicon. (B) Amplification of the junction between the 3’ end of PF23 and the 5’ end of m-scarlet in the rescue strain. Asterisks indicate non-specific bands. (C) Amplification of the junction between the 3’ end of PF23 and the 5’ end of neon green. (D) Amplification of the junction between the 3’ end of PF23 and aphvii in the pf23-4 null strain with an insertion in exon 1. (E) pf23-4 fails to amplify a band in exon 1 due to the aphvii insertion. Created with BioRender. (TIF) [file pgen.1011038.s004.tif]

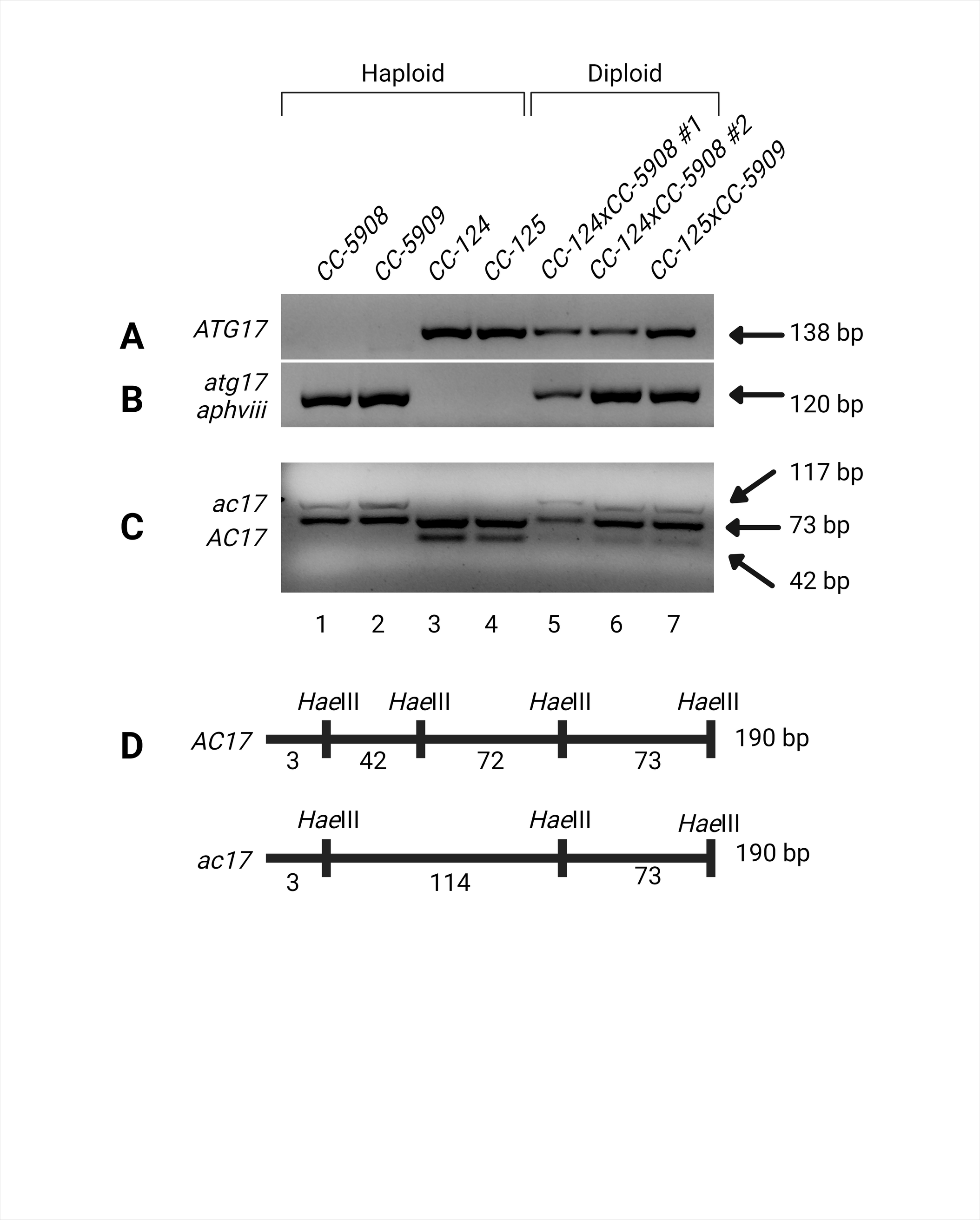

Supplement: S5 Fig — Haploid strains CC-5908 and CC-5909 carry an insertion in the ATG17 gene and a mutation in ac17 but are wild-type swimmers. CC-124 and CC-125 are wild-type at both loci. The three diploid strains are heterozygous for the atg17 and ac17 mutations. (A) PCR primers for the ATG17 gene [85] were used to detect the wild-type copy of the gene in CC-124, CC-125, and three diploid wild-type strains (lanes 3A-7A). No amplification is observed in CC-5908 and CC-5909 (lanes 1 & 2), since the aphviii paromomycin resistance gene is inserted into the atg17 gene and is too large to amplify with the condition used. (B) PCR primers were designed to detect the junction between the ATG17 gene and the aphviii insertion in the ATG17 gene. This junction is present in the CC-5908 and CC-5909 strains that carry the insertion (lanes 1B-2B), as well as the heterozygous diploids (lanes 5B-7B). (C) PCR primers were designed to amplify across the region containing the mutation in the AC17 gene [120]. Digestion with HaeIII generates different sized products in the ac17 containing strains (lanes 1C-2C) compared to CC-124 and CC-125 wild-types (lanes 3C-4C). The heterozygous diploid strains (5C-7C) generate both sets of digest products. (D) A map of the AC17 PCR product digested with HaeIII. Created with BioRender. (TIF) [file pgen.1011038.s005.tif]

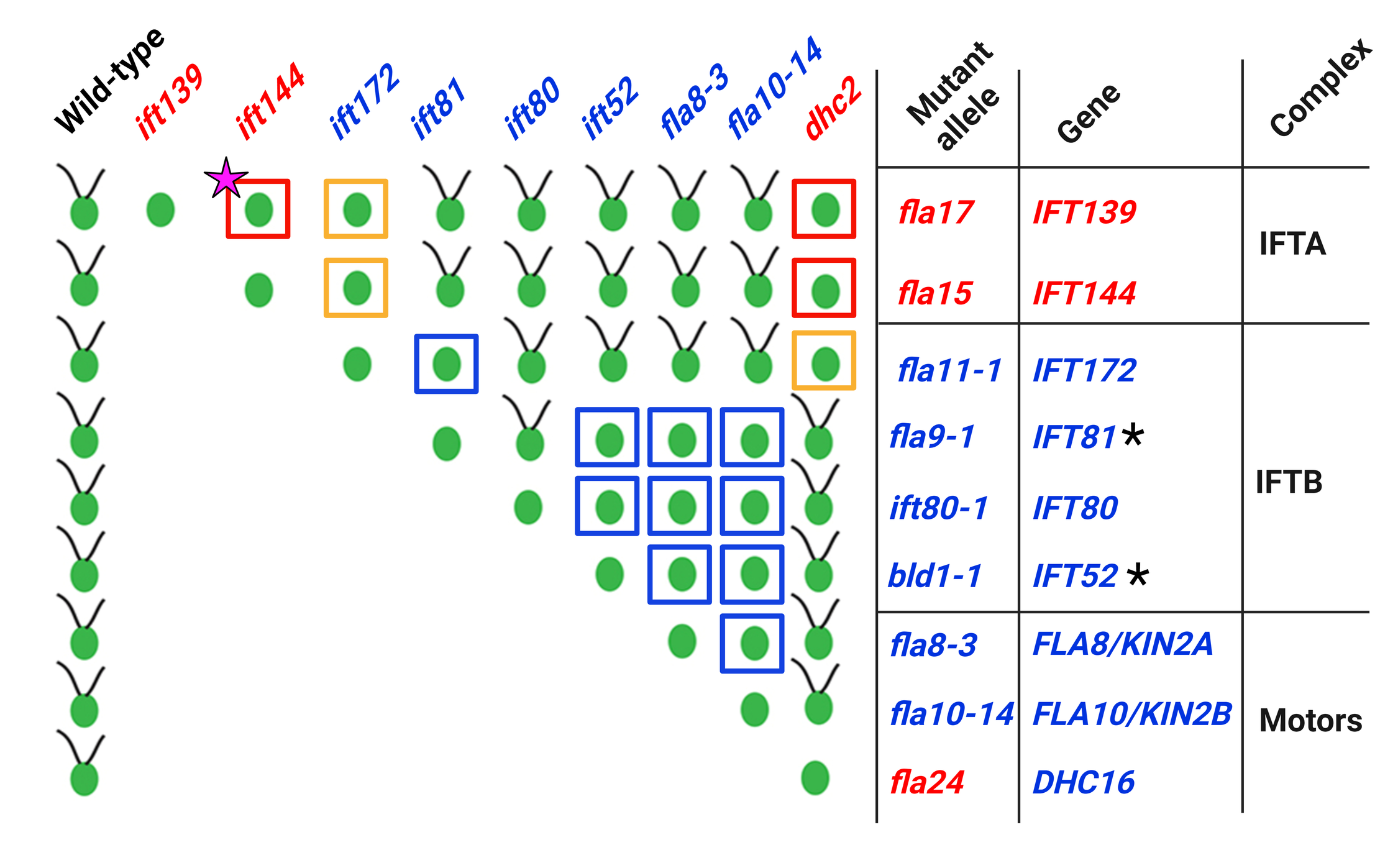

Supplement: S6 Fig — The ift mutants shown are temperature-sensitive or nonconditional (indicated by *). At the permissive temperature (21°C), these mutant strains assemble cilia and swim. After 12 hrs at the restrictive temperature (32°C), the strains are aciliate. The mutations are recessive; single heterozygotes assemble cilia at the restrictive temperature (left column). Several members of the same complex fail to complement as double heterozygotes (red squares represent IFTA, blue squares are IFTB). Interestingly, ift172 complements other components of the IFTB complex but fails to complement mutants of IFTA (yellow squares). The data were generated using methods described in Iomini et al. The original published SSNC combination of mutants ift144 and ift139 is highlighted by a pink star [82]. Created with BioRender. (TIF) [file pgen.1011038.s006.tif]

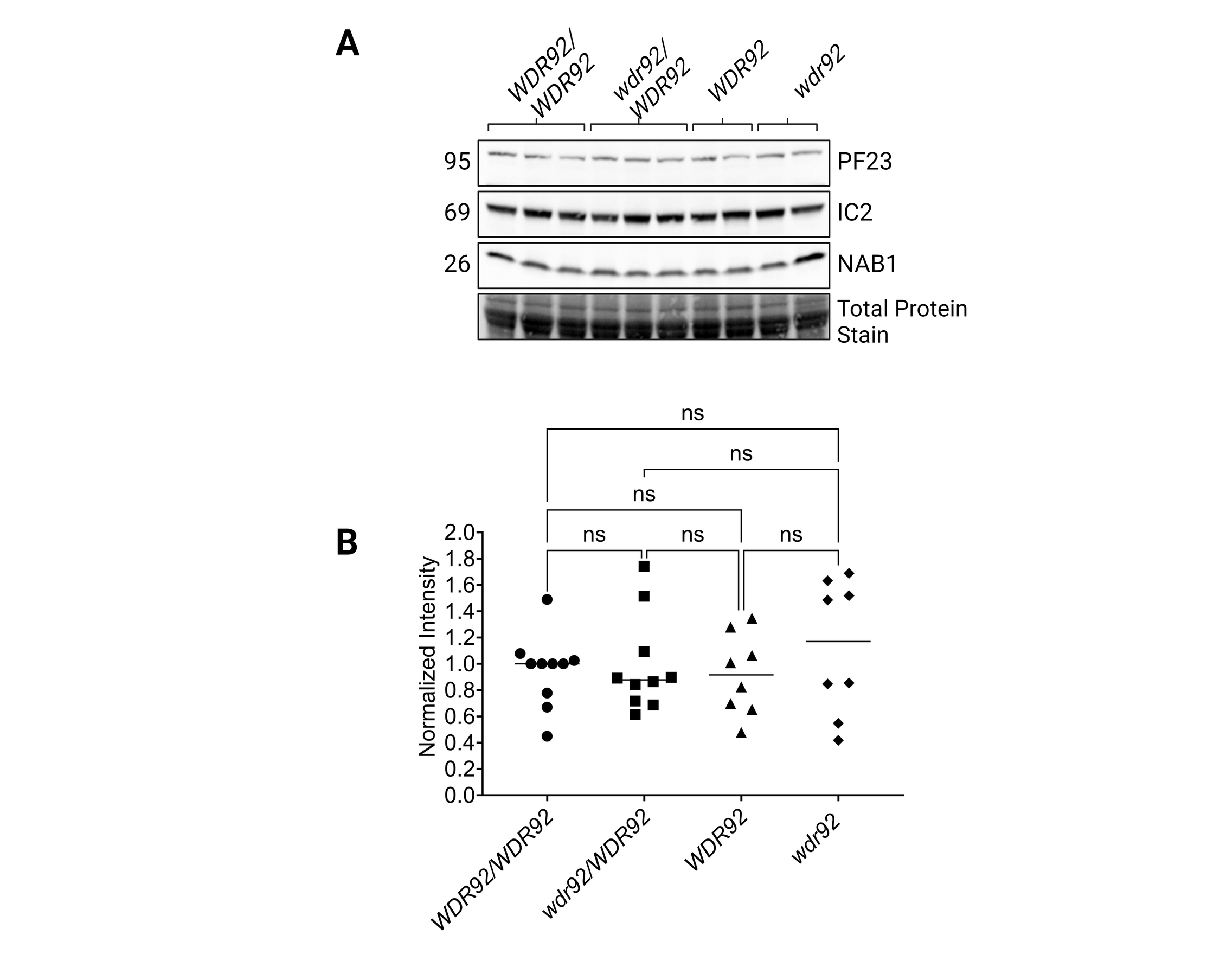

Supplement: S7 Fig — (A) Representative blot with 50 μg protein of the indicated strains. Each strain within the brackets indicates a biological replicate. Haploid strains with genotype wild-type WDR92 included CC-5908 and CC-124. IC2 and NAB1 are used as loading controls. Total protein stain was used for quantification. (B) Quantification of samples (biological and technical replicates). All samples were normalized to the mean of the wild-type diploids. A one-way ANOVA was used to assess statistical significance. ns: not significant. Created with BioRender. (TIF) [file pgen.1011038.s007.tif]
