# Supplementary material for: Gene dosage of independent dynein arm motor preassembly factors influences cilia assembly in Chlamydomonas reinhardtii
Source: PLoS Genet. 2024 Mar 18;20(3):e1011038. doi: 10.1371/journal.pgen.1011038 (PMC11020789; doi:10.1371/journal.pgen.1011038)
Supplement: S1 Table — Where indicated, the PCR product is digested with a restriction enzyme to distinguish between wild-type and mutant genotypes. ‘No band in mutant’ indicates the presence of an insertion that is too large to amplify during the extension time needed to amplify the PCR products with the same primers in wild-type. *Designed using the method for allele-specific primers (ASP) [117]. ** The mating-type primers can be run simultaneously to generate two bands that distinguish between both mating-types. (DOCX) [file pgen.1011038.s008.docx]

**Table S1: PCR primers to genotype strains used in this work.**

| **Strain** | **Locus** | **Forward Primer** | **Reverse Primer** | **Comments** |
| --- | --- | --- | --- | --- |
|  | | | | |
| *oda1* | Cre16.g666150 | TGCTTTAAACATCTGGCTTG | CTTGTGCAAACGGGTCAC | Mutant + wild-type *(Sac*I) |
| *oda2-921^* | Cre11.g476050 | CTGTACTGCATGACCTGGTC | TCTCCTCGTTCTTGGGGTAG | No band in mutant |
| *oda3-1* | Cre17.g703850 | CAGAAGAAGCTGCGTGAG | AGTCCTGCTCCATCTTGA | Mutant + wild-type (*Nru*I) |
| *oda4-1* | Cre09.g403800 | AAGACTCTGGAGGACAAGCT | AACCAGCATGGCATTGCCAC | Detects wild-type |
| *sup-pf1-1* | Cre09.g403800 | AGTGCCCAGTTTGCTCTTG | GACTTGAGCTCCGACAACTC | Mutant + wild-type (*Drd*I) |
| *oda5* | Cre01.g029750 | CAGTGTTGGGCGTTTGAAAA | ATCTACAACCCATCCCCACC | Mutant + wild-type (*BslI*) |
| *oda6-95* | Cre12.g506000 | CAGTTTGGCCGCTTTCCA | GTAGTCAACATCCTTGGGCC | Mutant + wild-type (*Hae*III) |
| *oda7-1* | Cre01.g029150 | ACAAAATGGACTCTACCGCA | ACTTCAGGTCCGCCAGT | No band in mutant |
| *ODA8* | Cre01.g043650 | CTGCAACTGGTCGTGAACT | CTGAGCGTCTGCAGATGC | Detects wild-type |
| *oda8* | Cre01.g043650 | TTATAATTAAATATAATAAACTACCTCGCCGCGTGCCACC | GTCACAGGCTCATCGTCAAG | Detects mutant |
| *ODA9* | Cre12.g536550 | GGCGATGAAGTCCAAAGC | CGTCGTCCCAGTACTTGAAG | Detects wild-type* |
| *oda9* | Cre12.g536550 | AGTGGGAGATCTACGACGAG | CCGCCTTCTTGCCGCA | Detects mutant* |
| *oda11-1* | Cre03.g145127 | CGGACCTGCGCCCAATAA | GCACGTCACCCTTCAGAAT | Detects mutant* |
| *oda12-2* | Cre12.g527750 | GTCCTCGATTTGTACCATGG | CATACACCGTTACAGCCTTG | No band in mutant |
| *oda14-f28* | Cre14.g617550 | GGTGGAGGACATGATTTGGG | CTCCTCCAGTGTGATGAAGC | No band in mutant |
| *pf14* | Cre06.g291700 | TACAACTAGTGCTCGCCTGA | GCGTATGTGTTGCCTCGTAC | Mutant + wild-type (*Mse*I) |
| *PF22* | Cre01.g001657 | CCCCATGCTCCCCTTGACTACA | GCCCTCGGGGCTGTCATCC | Detects wild-type* |
| *pf22-1* | Cre01.g001657 | GCCTGCCGGCTGTACGTATGA | CACCCCGCCGTCCAGCAG | Detects mutant* |
| *pf23-1* | Cre11.g467560 | CATTGGGTACTGGGATGATGGAAC | TGCAGCCGATGACTCCTTATAAGTTT | Detects mutant |
| *PF23* | Cre11.g467560 | ACACACACACATGCGCAAT | TCCGTAAGTAACTCCGTCGC | Detects wild-type |
| *WDR92* | Cre16.g672600 | ATGCTGCAAGGTGGTGTTG | CACACACCAGCAGTCCCT | Detects wild-type |
| *wdr92-2* | Cre16.g672600 | ATGCTGCAAGGTGGTGTTG | GCACCAATCATGTCAAGCCT | Detects mutant |
| *ida3* | Cre03.g205000 | ACTTGCTTTCTCACGGCACT | CATGAGACTCCTTCCGTGT | Mutant + wild-type (*SfcI*) |
|  | | | | |
| CC-124 | Mating-type minus (*MTD*) | GCATGGCCTCTTAATCAGAC | TTTGGAGTCCTCTCGTCAAG | ** |
| CC-125 | Mating-type plus (*MTA*) | TCTCCATGGGGTGTATCATC | TTTGGAGTCCTCTCGTCAAG | ** |
|  | | | | |
| *pf23-2* | Cre11.g467560 | TGTAAATGGAGGCGCTCGTTG | GATCGCCAAGCCCTGTTGC | Detects reverse insertion |
| *pf23-3* | Cre11.g467560 | TGTAAATGGAGGCGCTCGTTG | GATCGCCAAGCCCTGTTGC | Detects reverse insertion |
| *pf23-4* | Cre11.g467560 | TGTAAATGGAGGCGCTCGTTG | GTCCAGCGCGAACAGATAGG | Detects forward insertion |
| *PF23* exon 1 | Cre11.g467560 | GATCGCCAAGCCCTGTTGC | GTCCAGCGCGAACAGATAGG | Detects wild-type |
|  | | | | |
| *ATG17* | Cre16.g651350 | AAGAAGGAACACGCCAAAAC | GTAGGATCGGTTGCTGACG | Band in wild-type |
| *AC17/PHD2* | Cre03.g194200 | AGGCCCAGAAGA AGGAGATT | agccgggtccaagataacta | Mutant + wild-type (*Hae*III) |
| CC-5908/ CC-5909 | Cre16.g651350 | AAGAAGGAACACGCCAAAAC | GTATCGGAGGAAAAGCTGGC | *atg17; aphviii* junction |
| *PF23* exon 1-2 | Cre11.g467560 | TTCTCAAAGTCAACAGCCCG | ATAGGCGCGGTCAATGGAG | cDNA primers to detect exon 1-2 junction |
| *ODA3* exon 5-6 | Cre17.g703850 | CAACATCAAGCGGAAGAAGG | TGATGTAGTCACGGTCCAC | cDNA primers to detect exon 5-6 junction |
| **PF13 deletion mapping** | | | | |
| *PF13* 3’UTR | Cre09.g411450 | CGCAGTGATGAATGGCAGAG | CGCCGGAATGATGGTCTTAC | *pf13-1* deletion verification. The deletion is not observed in wild-type. |
| *PF13* exon 10 | Cre09.g411450 | TCACACTTCCTGTCGTACCC | CTGATCCCGACCCATCACAT |  |
| *PF13* exon 7 | Cre09.g411450 | GTACAAGGGCACGGAGGG | GGTCTTGGCTCCCGGAAG |  |
| *PF13* exon 3 | Cre09.g411450 | GCCGTCATCAAAACCAAGGT | TGTCACAGGTGCAGATGTTG |  |
| Uridine hydrolase | Cre09.g411500 | CCGCACATCAACGACCTTAC | GTGCTCCTTTAACGGCTACC |  |
| Tyrosine kinase | Cre09.g411525 | CAAACATGACAGCTCCGGAG | GCTGAACCGTAACCGTGAC |  |
| Myb domain | Cre09.g411600 | GTGGTCTGAGCCTCCCTTAC | AGGTCAAGGGTCATAGGTGC |  |
| TAZ ring finger | Cre09.g411633 | TCGAACTGGCTGAGACTGG | AAGTTTGCGCACTGATGAGG |  |
| Unknown | Cre09.g411666 | CGGCGACTTTTATGGCATGG | CGCACGCATACATACGCAT |  |
| Unknown exon 3a | Cre09.g411700 | CAAACCTCCTCCAACTGCAC | CGCCATAGTCTCCGAAGTCA |  |
| Unknown exon 3b | Cre09.g411700 | CGCCAGAGTTTGAGTCGTAC | CAGATTGTGCCGTACGTCAA |  |
| Unknown exon 2-intron 2 | Cre09.g411700 | TCCCAAATTATCCGCCGTTG | GCATTTTAGGGGCATGGAGG |  |
| Unknown exon 1 | Cre09.g411700 | GCTGCGGTAGTTAGAGCCTT | AGATAAATGATGCAGAGCGGC |  |
| **PF13 recombineering primers** | | | | |
| *PF13* | Cre09.g411450 | CGGCGGTGCTGCGGCCGCGGCTGAATCGCGAGTTGGCTATGGAGTTGGATGGAGATCTGGGTGGCTCCG | TTATGTTATTTTGTGTTGTGTTGTGTGTGCGTCGCTGTGTCTTGAGTCTT GAAGATCCTTTGATCTTTTCTACGGG | Recombineering homology primers |
| *PF13* 5’ end | Cre09.g411450 | CTTTGAGAGAGGGCCAAGGG | GGTCATCTCCTGCAGCAGTT | BAC verification |
| *PF13* 3’ end | Cre09.g411450 | CTGAACCCTCGTCTCGATCG | CTCCATAGCCAACTCGCGAT | BAC verification |
| *PF13:YFP-FLAG-TG* | Cre09.g411450 | CTGAACCCTCGTCTCGATCG | CAGATCAGCTTCAGGGTCAG | PF13-YFP 3’ junction verification |
| **PF23 recombineering primers** | | | | |
| *PF23* | Cre11.g467560 | CTGGCGGGAGAGGGGCGGGCGAGGGCCTGGCGGATGATGACGTGGACGACGGAGATCTGGGTGGCTCCG | AAGGTAAATGATATCAGGGGATGGGCAAAATCCTGCAGCTTTAAAGCATTGAAGATCCTTTGATCTTTTCTACGGG | Recombineering homology primers |
| *PF23* 5’ end | Cre11.g467560 | TCAAAGAGCAAGGCCGATGT | CCCACCTTGAAGAGCGTGAA | BAC verification |
| *PF23* 3’ end | Cre11.g467560 | GGGTAGAGGCTGGTGGATTG | AGTCGTCCACGTCATCATCC | BAC verification |
| *PF23:m-scarlet-FLAG-TG* | Cre11.g467560 | GGGTAGAGGCTGGTGGATTG | GTACATGAACTGAGGGGACAG | *PF23*-m-scarlet 3’ junction verification |
| *PF23:neon green-FLAG-TG* | Cre11.g467560 | GGGTAGAGGCTGGTGGATTG | GTGGACTTCAGGTTCAGCTC | *PF23*-neon green 3’ junction verification |
