# Supplementary material for: Gene dosage of independent dynein arm motor preassembly factors influences cilia assembly in Chlamydomonas reinhardtii
Source: PLoS Genet. 2024 Mar 18;20(3):e1011038. doi: 10.1371/journal.pgen.1011038 (PMC11020789; doi:10.1371/journal.pgen.1011038)
Supplement: S2 Table — The protein, gene, and mutant names are in columns 1–3. The normalized log2 values from the average of three biological replicates are presented. Proteins in the MIA complex, radial spokes, N-DRC, Central apparatus, and MIPs are presented to show small or no changes. (DOCX) [file pgen.1011038.s009.docx]

**Table S2: Raw Log2 values from TMT Mass Spectrometry**

| **Protein** | **Gene** | **Mutant** | **Log_2_ Normalized Intensity**  **Wild-Type Average**  **N=3** | **Log_2_ Normalized Intensity**  ***pf23-4; cnk11* Average**  **N=3** | **Log_2_ Normalized Intensity**  ***cnk11* Average**  **N=3** |
| --- | --- | --- | --- | --- | --- |
| **Tubulin** | | | | | |
| Tubulin α | *TUA1*  *TUA2* |  | 7.92 | 9.04 | 6.92 |
| Tubulin β | *TUB1*  *TUB2* |  | 6.55 | 6.35 | 8.07 |
| **ODA** | | | | | |
| α-HC | *DHC13* | *oda11* | 11.2 | -- | 10.5 |
| β-HC | *DHC14* | *oda4* | 11.6 | -- | 10.6 |
| γ-HC | *DHC13* | *oda2* | 12.8 | -- | 10.8 |
| IC1 | *DIC1* | *oda9* | 11.5 | -- | 10.8 |
| IC2 | *DIC2* | *oda6* | 12.4 | -- | 11.5 |
| LC1 | *DLU1* |  | 11.8 | -- | 11.5 |
| LC2 | *DLT2* | *oda12* | 11.2 | 8.9 | 10.8 |
| LC3 | *DLX1* |  | 14.2 | 5.5 | 12.1 |
| LC4 | *DLE1* |  | 14.8 | 14.4 | 13.6 |
| LC5 | *DLX2* |  | 12.6 | -- | 11.7 |
| LC6 | *DLL2* | *oda13* | 11.7 | 5.1 | 10.9 |
| LC9 | *DLT1* |  | 11.5 | 2.5 | 12.1 |
| LC10 | *DLL3* |  | 14.8 | -- | 12.7 |
| **ODA and IDA light chains** | | | | | |
| LC7a | *DLR1* | *oda15* | 8.84 | 3.93 | 8.61 |
| LC7b | *DLR2* |  | 3.44 | - | 3.20 |
| LC8 | *DLL1* | *fla14* | 12.0 | 11.67 | 10.57 |
| **IDAI1/f** | | | | | |
| I1/f α | *DHC1* | *pf9/ida1* | 11.6 | -- | 10.9 |
| I1/f β | *DHC10* | *ida2* | 10.2 | -- | 9.8 |
| IC138 | *DIC4* | *bop5* | 11.2 | -- | 10.3 |
| IC140 | *DIC3* | *ida7* | 9.96 | -- | 9.5 |
| IC97 | *DII6* |  | 10.3 | -- | 9.5 |
| FAP120 | *DII7* |  | 7.9 | -- | 9.06 |
| TCTEX1 | *DLT3* |  | 9.4 | 5.5 | 7.6 |
| TCTEX2b | *DLT4* |  | 10.9 | 8.8 | 10.3 |
| **Monomeric IDAs** | | | | | |
| DHC2 |  |  | 10.9 | 1.9 | 10.8 |
| DHC3 |  |  | 9.8 | -- | 8.9 |
| DHC4 |  |  | 8.4 | 1.13 | 7.8 |
| DHC5 |  |  | 12.1 | 0.8 | 11.2 |
| DHC6 |  |  | 11.7 | 1.95 | 10.9 |
| DHC7 |  |  | 11.1 | -- | 10.6 |
| DHC8 |  |  | 10.4 | 1.19 | 9.5 |
| DHC9 |  | *ida9* | 10.8 | 0.62 | 10.2 |
| DHC11 |  |  | 9.97 | -- | 10.1 |
| p28 | *DII1* | *ida4* | 11.2 | -- | 11.3 |
| actin | *DII4* | *ida5* | 10.5 | 3.7 | 9.5 |
| centrin | *DLE2* | *vfl2* | 10.0 | 10.0 | 10.3 |
| p44 | *DII3* |  | 8.87 | 8.93 | 105 |
| p38 | *DII2* |  | 7.90 | 6.14 | 7.1 |
| DHC12 | not present | | | | |
| **IDA associated** | | | | | |
| FAP44 |  |  | 8.87 | 10.5 | 8.83 |
| FAP244 |  |  | 9.98 | 1.24 | 9.71 |
| FAP43 |  |  | 8.16 | 9.9 | 7.3 |
| CCDC146 |  | *mbo2* | 8.03 | 10.08 | 7.96 |
| FAP57 |  | *bop2/ida8* | 8.58 | 10.43 | 7.78 |
| FAP263 |  |  | 8.79 | 7.54 | 8.47 |
| **ODA Docking Complex** | | | | | |
| DC1 | *DCC1* | *oda3* | 8.9 | 10.1 | 8.5 |
| DC2 | *DCC2* | *oda1* | 9.4 | 10.5 | 8.7 |
| DC3 | *DLE3* | *oda14* | 8.9 | 9.7 | 7.8 |
| **Modifiers of Inner Arms** | | | | | |
| FAP73 |  | *mia2* | 9.76 | 10.6 | 9.2 |
| FAP100 |  | *mia1* | 9.9 | 11.3 | 8.93 |
| **Radial Spokes** | | | | | |
| FAP61 |  |  | 7.7 | 11.03 | 8.7 |
| FAP91 |  | *rsp18* | 7.8 | 9.8 | 7.3 |
| FAP184 |  |  | 9.7 | 11.4 | 8.5 |
| **Nexin-Dynein Regulatory Complex** | | | | | |
| DRC1 |  | *pf3* | 10.4 | 12.03 | 10.13 |
| DRC2 |  | *ida6* | 9.27 | 10.3 | 8.3 |
| **Central Apparatus** | | | | | |
| Hydin |  |  | 7.5 | 9.3 | 7.1 |
| CPC1 |  |  | 7.9 | 6.7 | 8.3 |
| **Microtubule Inner Protein** | | | | | |
| FAP166 |  |  | 10.6 | 11.8 | 10.1 |
| RIB72 |  |  | 9.2 | 11.2 | 8.8 |
| RIB43a |  |  | 8.8 | 10.9 | 8.9 |
| PACRG |  | *pacrg* | 8.45 | 9.85 | 7.24 |
