# Supplementary material for: Gene dosage of independent dynein arm motor preassembly factors influences cilia assembly in Chlamydomonas reinhardtii
Source: PLoS Genet. 2024 Mar 18;20(3):e1011038. doi: 10.1371/journal.pgen.1011038 (PMC11020789; doi:10.1371/journal.pgen.1011038)
Supplement: S3 Table — Each mutation is classified by the IFT complex in which it has been found. The human ortholog of each Chlamydomonas IFT gene is listed. **These strains produce a mutant protein with partial function at the permissive temperature. #Null at the restrictive temperature. (DOCX) [file pgen.1011038.s010.docx]

| **Category** | **Mutant** | **Human**  **Ortholog** | **PCD Gene** | | **DNA or animo acid change** | **Type of allele** | **Ref.** |
| --- | --- | --- | --- | --- | --- | --- | --- |
| **IFTA** | *fla15-1* | *WDR19* | No | C1283R | | Altered**/ Null^#^ | [2] |
|  | *fla17-1* | *TTC21A* | No | In-frame deletion of 3 exons | | Altered**/ Null^#^ | [2] |
|  |  |  |  |  | |  |  |
| **IFTB** | *fla11-1* | *IFT172* | No | L1615P | | Altered**/ Null^#^ | [3] |
|  | *ift81* | *IFT81* | No | 3’ splice site of exon 7 | | Altered**/  Null^#^ | [4] |
|  | *ift80* | *IFT80* | No | W🡪X | | Null | [5] |
|  | *bld1-1* | *IFT52* | No | Insertion | | Null | [6] |
|  |  |  |  |  | |  |  |
| **IFT**  **Motors** | *fla10-14* | *KIF3A* | No | E24K | | Altered**/  Null^#^ | [7] |
|  | *fla8-3* | *KIF3B* | No | E24G | | Altered**  Null^#^ | [8] |
|  | *fla24-1* | *DYNC2H1/DHC1b* | No | L3243P | | Altered**  Null^#^ | [8] |

**Table S3: Mutations in temperature-sensitive IFT mutants in *Chlamydomonas***
